# AgriX-SENet: Squeeze-and-Excitation-based deep learning framework for explainable plant disease detection in sustainable agriculture

**DOI:** 10.3389/fpls.2026.1819042

**Published:** 2026-07-29

**Authors:** Shreyta Raj, Prashant Johri, Vishwadeepak Singh Baghela, Arfat Ahmad Khan, Muhammad Shoaib Ayub, Insoo Koo

**Affiliations:** 1 School of Computer Science and Engineering, Galgotias University, Greater Noida, India; 2 Department of Computer Science, College of Computing, Khon Kaen University, Khon Kaen, Thailand; 3 Department of Electrical, Electronic and Computer Engineering, University of Ulsan, Ulsan, Republic of Korea

**Keywords:** deep learning, DenseNet121, explainable tool, feature extraction, plant disease, Squeeze-and-Excitation, sustainable agriculture

## Abstract

**Introduction:**

Timely and accurate detection of plant diseases is essential for ensuring global food security and supporting sustainable agriculture. Conventional diagnostic approaches, such as manual inspection and laboratory testing, are often time-consuming, labor-intensive, and impractical for large-scale or remote agricultural environments. Although deep learning models, particularly Convolutional Neural Networks (CNNs), have significantly improved automated plant disease classification, they often lack interpretability and struggle to generalize under diverse field conditions.

**Methods:**

This study proposes AgriX-SENet, an explainable deep learning framework that integrates Squeeze-and-Excitation (SE) blocks with a DenseNet121 backbone to enhance disease classification performance. The SE blocks recalibrate channel-wise feature responses to emphasize disease-relevant information while suppressing background noise. To improve model transparency, Grad-CAM, SHAP, and LIME were incorporated to provide visual and feature-level explanations of the model’s predictions. The framework was trained and evaluated using the Plant Pathology 2020 dataset containing four classes: healthy, rust, scab, and multiple diseases.

**Results:**

AgriX-SENet achieved a training accuracy of 97.47% and a validation accuracy of 95.07%, outperforming fourteen state-of-the-art deep learning models. The classification report demonstrated high precision and recall across most disease categories, although the scab class exhibited comparatively lower recall, indicating an opportunity for further improvement. The explainability analyses consistently showed that the model focused on pathologically relevant regions of leaf images, validating the reliability of its predictions.

**Discussion:**

The proposed AgriX-SENet framework effectively combines high classification performance with model interpretability, addressing a key limitation of existing CNN-based plant disease detection systems. Its ability to provide accurate and explainable predictions makes it a promising solution for scalable agricultural diagnostics. Future work will focus on improving classification performance for challenging disease categories and optimizing the framework for deployment on mobile and edge computing devices to enable real-time field applications.

## 1 Introduction

Agriculture is undergoing a digital revolution driven by the urgent need to feed an ever-growing population, projected to exceed 9 billion by 2050, amidst diminishing arable land, volatile climates, and increasing demand for resource-efficient farming (UN Population Division, 2009). At the heart of this transformation lies sustainable agriculture, a holistic approach aimed at maximizing productivity while preserving environmental quality, economic viability, and social equity. However, one of the most persistent threats to sustainable crop production is plant disease, which can silently devastate crops, cause economic losses, and destabilize food supply chains (Naveed et al., 2025; Oumaima et al., 2025).

According to the Food and Agriculture Organization (FAO), plant diseases contribute to the loss of over 30–40% of global agricultural production annually (About | Plant Production and Protection). These diseases often spread rapidly and are influenced by subtle environmental conditions, making timely detection and diagnosis a critical requirement. Conventional methods rely heavily on manual inspection and laboratory testing, which, while effective in controlled environments, are inherently limited in scalability, response time, and accuracy—especially in remote or resource-constrained regions (Tripathi et al., 2024; Anand et al., 2025).

In recent years, deep learning (DL), particularly Convolutional Neural Networks (CNNs), has demonstrated remarkable success in automating visual recognition tasks, including plant disease identification (Khan et al., 2025; Papazoglou et al., 2025). Leveraging large-scale image datasets, these models can learn intricate patterns and distinguish between subtle symptoms across various crops and pathogens. Despite their high accuracy, however, CNN-based approaches are often criticized for their lack of transparency and interpretability (Nobel et al., 2024; Polly and Devi, 2024). In agriculture, where domain expertise is essential and decisions impact both livelihoods and ecosystems, explainability becomes just as important as performance.

The adoption of AI in agriculture hinges on user trust—farmers, agronomists, and policymakers need to understand how a model arrives at a particular conclusion, especially in high-stakes decisions such as disease treatment (Aijaz et al., 2025; Mamun et al., 2026). Most deep learning models operate as “black boxes”, providing outputs without clear rationales. This lack of interpretability raises concerns about reliability, accountability, and adaptability across different contexts and crop varieties.

Furthermore, many existing models struggle in complex environments, where lighting, background clutter, leaf occlusion, or co-infections may introduce noise. These challenges necessitate the development of architectures that not only focus on relevant visual cues but also provide interpretable insights to end-users (George et al., 2025; Mamun et al., 2025).

To address these limitations, attention mechanisms have emerged as a powerful tool in deep learning. Among them, the Squeeze-and-Excitation (SE) block is particularly promising. First introduced in SENet, SE blocks allow CNNs to adaptively recalibrate channel-wise feature responses by modeling interdependencies between channels. This means the network can emphasize more informative features (e.g., disease patterns on leaves) while suppressing irrelevant ones (e.g., background elements). The integration of SE blocks leads to a more focused and context-aware model, which is especially beneficial in agricultural scenarios where symptoms may appear as minor discolorations or textural anomalies. Despite its proven success in general computer vision tasks, SE-based architectures remain underexplored in plant disease detection, particularly in combination with explainable AI techniques (Peyal et al., 2024; Shanmugam et al., 2025). Techniques such as Gradient-weighted Class Activation Mapping (Grad-CAM), Layer-wise Relevance Propagation (LRP), and Local Interpretable Model-Agnostic Explanations (LIME) generate visual or textual explanations of a model’s predictions. When combined with attention mechanisms like SE, they offer a pathway to develop both accurate and explainable systems—essential for real-world agricultural deployment. To bridge this gap, we propose AgriX-SENet, a deep learning framework that fuses the power of Squeeze-and- Excitation attention modules with interpretability tools to enable explainable, robust, and scalable plant disease detection. AgriX-SENet is designed with the following goals:

- Enhanced Feature Discrimination: SE blocks are embedded within a CNN backbone to amplify disease- relevant feature maps and suppress noisy background information.
- Transparent Decision-Making: The framework incorporates Grad-CAM for class-specific localization maps and LIME for local feature attribution, enabling users to visualize what parts of the input image influenced the prediction.
- Generalizability and Efficiency: The architecture is lightweight and adaptable to various plant species and disease types, allowing deployment on mobile or edge devices for real-time field diagnostics.
- Alignment with Sustainability Goals: By enabling early and accurate disease detection, AgriX-SENet supports targeted intervention strategies, reducing over-reliance on chemical pesticides, lowering input costs, and contributing to environmentally responsible farming.

This research presents AgriX-SENet, a deep learning framework for explainable plant disease detection tailored to the goals of sustainable agriculture. The key contributions of this work are outlined as follows:

- Study proposes a deep learning model that integrates Squeeze-and-Excitation (SE) blocks to enhance feature extraction and focus on disease-relevant patterns in plant images.
- The model incorporates Grad-CAM and LIME to provide visual and interpretable explanations, improving transparency and user trust.
- AgriX-SENet is optimized for low-resource environments, making it suitable for deployment on mobile and edge devices for real-time field diagnostics.
- By enabling early and explainable disease detection, AgriX-SENet contributes to reduced pesticide use, precision interventions, and overall sustainable farming practices.

The remainder of this paper is organized as follows: Section 2 presents a review of related work in plant disease detection using deep learning and attention mechanisms. Section 3 describes the proposed AgriX-SENet architecture and the methodology for model training and evaluation. Section 4 discusses the experimental setup,

datasets used, and performance metrics. Section 5 analyzes the results, interpretability outputs, and implications for sustainable agriculture. Finally, Section 6 concludes the paper and outlines directions for future work.

## 2 Related work

This work (Qushtom et al., 2025) has combined convolutional neural networks (CNNs) with Explainable AI (XAI) to enhance wheat disease detection. Using data from Kotli, Azad Kashmir, the model achieved up to 100% accuracy with improved stability and faster convergence. XAI methods like LIME and SHAP provided insights into key features influencing predictions. This approach offers both high accuracy and interpretability, supporting effective and sustainable crop disease management.

In their study, the authors present a deep learning model using transfer learning for cassava disease detection based on field images collected in Tanzania. The model identifies three diseases and two types of pest damage with high accuracy—up to 98% for specific classes and 93% overall on unseen data. This approach demonstrates the potential of scalable, low-cost image recognition techniques for real-time plant disease detection, particularly in support of food security efforts in sub-Saharan Africa.

The present model (Rashid et al., 2025) of a transfer learning-based deep learning model for cassava leaf disease detection using field images from Tanzania. The model classifies three diseases (CBSD, CMD, BLS), two pest damages (RMD, GMD), and healthy leaves, achieving up to 98% class-wise and 93% overall accuracy on unseen data. Image preprocessing and augmentation improved generalization. This scalable and low-cost solution enables real-time, mobile-based disease detection, supporting food security in sub-Saharan Africa.

This study (Savaş, 2024) explores deep learning for palm disease detection in smart agriculture, aiming to improve robustness and generalization over prior work. A two-stage approach is used: first, transfer learning with fine- tuned models like MobileNetV2, ResNet, ResNetRS50, and DenseNet121, achieving over 92% accuracy; second, an ensemble learning method enhances performance, with the DELM1 model reaching a 99% ROC AUC score. The framework combines multiple disease and pest classes across datasets, offering a comprehensive and effective classification system for real-world crop monitoring.

This study (Attri et al., 2025) presents a TinyML-based model using MobileNetV2 for real-time detection of apple and mango leaf diseases on edge devices. The model achieved 93.1% accuracy and outperformed ResNet-50, VGG-16, and AlexNet. Its lightweight design makes it ideal for remote, low-resource settings, supporting sustainable farming. Future work aims to expand crop coverage, integrate sensor data, and validate performance through field trials.

This paper (Piccialli et al., 2025) presents AGRIFOLD, a Federated Learning (FL) framework for privacy-preserving training of a lightweight CNN across diverse agricultural datasets. By integrating Efficient Channel Attention into VGG16, the model boosts accuracy and interpretability using heatmaps. Among various aggregation methods, SCAFFOLD performed best. AGRIFOLD’s optimized design supports deployment on edge devices and includes an NLP-based recommender for disease treatment. Tested on 12 datasets across 9 disease classes, it demonstrates strong potential for real-world, sustainable agriculture.

Rozaqi and Sunyoto (2020) (Rozaqi and Sunyoto, 2020) implemented a tailored CNN model to identify early and late blight in potato leaves, as well as healthy samples. Using a 70:30 training-test split, the model reached 97% accuracy in training and 92% in testing, with a batch size of 20 over 10 epochs. Likewise (Khobragade et al., 2022), introduced a CNN-based solution for classifying potato leaf images into three categories—healthy, early blight, and late blight—using a dataset comprising 5,162 original and 82,592 augmented images, achieving 98.07% accuracy. In (Singla et al., 2022), a deep learning model was employed to assess the severity of rice hispa infestations in Punjab, India, delivering 98.86% accuracy for binary classification and 98.6% for multi-class scenarios. Jung et al. (2023) (Jung et al., 2023) proposed a plant disease detection framework that used a stepwise classification strategy along with five pre-trained CNN models, attaining 97.09% accuracy on the test dataset. Mac et al. (2024) (Mac et al., 2024) enhanced greenhouse navigation and tomato disease diagnosis by integrating a fuzzy control system with deep learning. They used a DCGAN to generate synthetic leaf images, increasing the ResNet-152 model’s performance to 99.69%, compared to 97.07% on the original data. Prasad et al. (2024) (Prasad et al., 2024) adopted both CNN and a modified VGG16-based deep convolutional neural network (DCNN) for classifying grape leaf diseases, achieving 99.18% accuracy in training and 99.06% in testing, with comprehensive metric evaluations such as the F1-score. Rahman et al. (2024) (Rahman et al., 2024) developed a CNN-based classifier for tea leaf diseases in Sylhet, Bangladesh, capable of distinguishing red rust, brown blight, grey blight, and healthy leaves with 96.65% accuracy, confirmed through lab validation. Lastly, Kini et al. (2024) (Kini et al., 2024) utilized transfer learning with CNNs to identify black pepper leaf diseases—including anthracnose, slow wilt, early and late phytophthora, and yellowing—using a field-verified dataset. Their best-performing model, ResNet18, achieved a remarkable 99.67% accuracy with a learning rate of 0.001. Collectively, these studies demonstrate the strong potential of deep learning and image analysis in accurately detecting and classifying crop diseases across a wide range of plant species and conditions.

Panchbhai et al. (2024) developed CAS-MODMOBNET, a lightweight MobileNetV2 variant for cashew disease detection. The model optimizes feature extraction with fewer parameters and a 3×3 average pooling layer. However, its performance on other datasets is yet to be evaluated. Raj Kumar et al. (2023) introduced a compact CNN to detect early and late blight in tomatoes and potatoes, reducing parameters by 70%. While efficient, it may miss complex features, affecting accuracy. Arya and Mishra (2024) proposed a hybrid model by integrating an Inception module into MobileNetV2, using transfer learning to improve feature learning and accuracy. Alkanan and Gulzar (2024) enhanced MobileNetV2 with additional layers to improve corn disease classification, strengthening its ability to detect detailed features. Sutaji et al (Sutaji and Yıldız, 2022). created an ensemble of MobileNetV2 and Xception to balance mobile efficiency and rich feature extraction. The model achieved improved accuracy using transfer learning and fine-tuning.

Based on the literature review provided, the following research gaps emerges:

- Despite the proven benefits of SE blocks in enhancing feature representation by emphasizing important channels, most existing models use conventional CNNs or variants like MobileNetV2, ResNet, and VGG without explicit SE integration.
- While some models (e.g (Qushtom et al., 2025) (Piccialli et al., 2025)) use XAI tools like LIME and SHAP, there’s no framework that combines attention mechanisms (like SE) with deep explainability to provide both intrinsic and *post-hoc* explanations.
- Most prior works are focused on single crops or limited disease types, with limited generalization across diverse datasets or field conditions.

Based on the literature review and identified research gaps, here is a well-defined research objective;

To develop a Squeeze-and-Excitation (SE)-based deep learning framework (AgriX-SENet) that enables accurate, explainable, and efficient detection of plant diseases across multiple crop types, with the goal of supporting sustainable agriculture through edge-compatible deployment and real-world applicability.

## 3 Materials and methodology

### 3.1 Dataset acquisition and characteristic

The Plant Pathology 2020 dataset was developed through a collaborative effort between Washington State University (WSU) and the United States Department of Agriculture (USDA) with the aim of enabling machine learning-based diagnosis of plant diseases under real-world field conditions (Plant Pathology 2020 - Fgvc7). Data collection was conducted in commercial apple orchards located in Washington State, a major hub for apple production in the United States. The imaging process was performed under natural lighting conditions and captured a range of environmental variability, including differences in leaf orientation, shadows, background clutter, and partial occlusions. This approach ensured that the dataset reflects the complexity and diversity encountered in operational agricultural settings, thereby enhancing its utility for developing robust, deployable computer vision models.

The dataset encompasses multiple apple cultivars to introduce genetic and morphological variability in the leaf structure and disease manifestation. Annotation was carried out by trained plant pathologists and agricultural extension professionals using a standardized labeling protocol. Labels were assigned based on characteristic visual features such as lesion color, size, shape, distribution, and progression stage. In cases where disease symptoms were ambiguous or where multiple infections co-occurred on a single leaf, a distinct “multiple_diseases” category was used.

The final dataset comprises 7284 labeled images divided into four mutually exclusive classes: healthy, rust, scab, and multiple_diseases. Rust is typically caused by fungal pathogens of the genus Gymnosporangium and appears as orange or yellow spots, whereas scab is induced by Venturia inaequalis and manifests as dark, velvety lesions often associated with leaf deformation. The dataset provides comprehensive coverage of disease symptoms and maintains a balanced class distribution, with 1,821 images in each category (healthy, rust, scab, and multiple diseases). This balanced distribution helps minimize class-specific bias during model training and evaluation. This class distribution presents additional challenges for model training, particularly for deep learning frameworks that are sensitive to data imbalance. The characteristics of leaf conditions in the plant pathology 2020 dataset is shown in **Table 1**. It was publicly released as part of the Kaggle Plant Pathology 2020—FGVC7 competition, under an academic- friendly license that encourages open research and model development. The image count in terms of class wise counting is unveiled as follows (**Table 2**):

**Table 1:** Characteristics of leaf conditions in the plant pathology 2020 dataset.

Class	Pathogen	Visual symptoms	Color	Lesion shape/pattern	Severity variation
Healthy	None	No visible signs of infection or damage	Uniform green	None	N/A
Rust	*Gymnosporangium* spp. (fungal)	Small raised pustules or spots, often powdery or spore-like in texture	Yellow to orange	Circular to oval, scattered on upper/lower leaf surface	Mild to severe
Scab	*Venturia inaequalis*(fungal)	Dark, velvety lesions, sometimes sunken, often deforming the leaf	Olive green to dark brown/black	Irregular, blotchy lesions, often on leaf margin	Mild spotting to extensive necrosis
Multiple Diseases	Rust + Scab or ambiguous symptoms	Combination of rust and scab features, or overlapping/uncertain symptom presentation	Mixed (e.g., orange + brown)	Mixed lesion types or unclear patterns	Variable

**Table 2:** Class category and number of images in the dataset.

S no	Class category	Number of images
1	Healthy	1821
2	Rust	1821
3	Scab	1821
4	Multiple Diseases	1821

**Figure 1:**
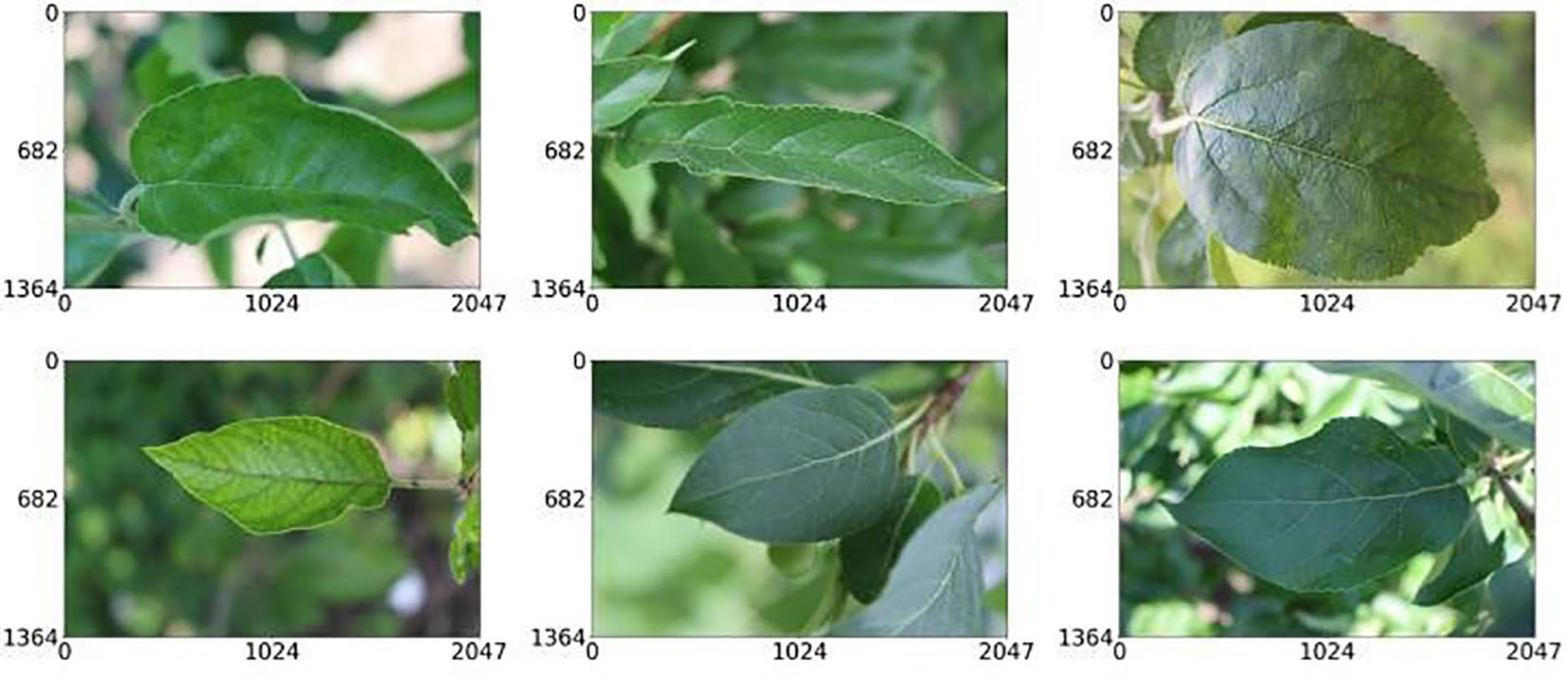
Visualization of healthy leaf class.

**Figure 2:**
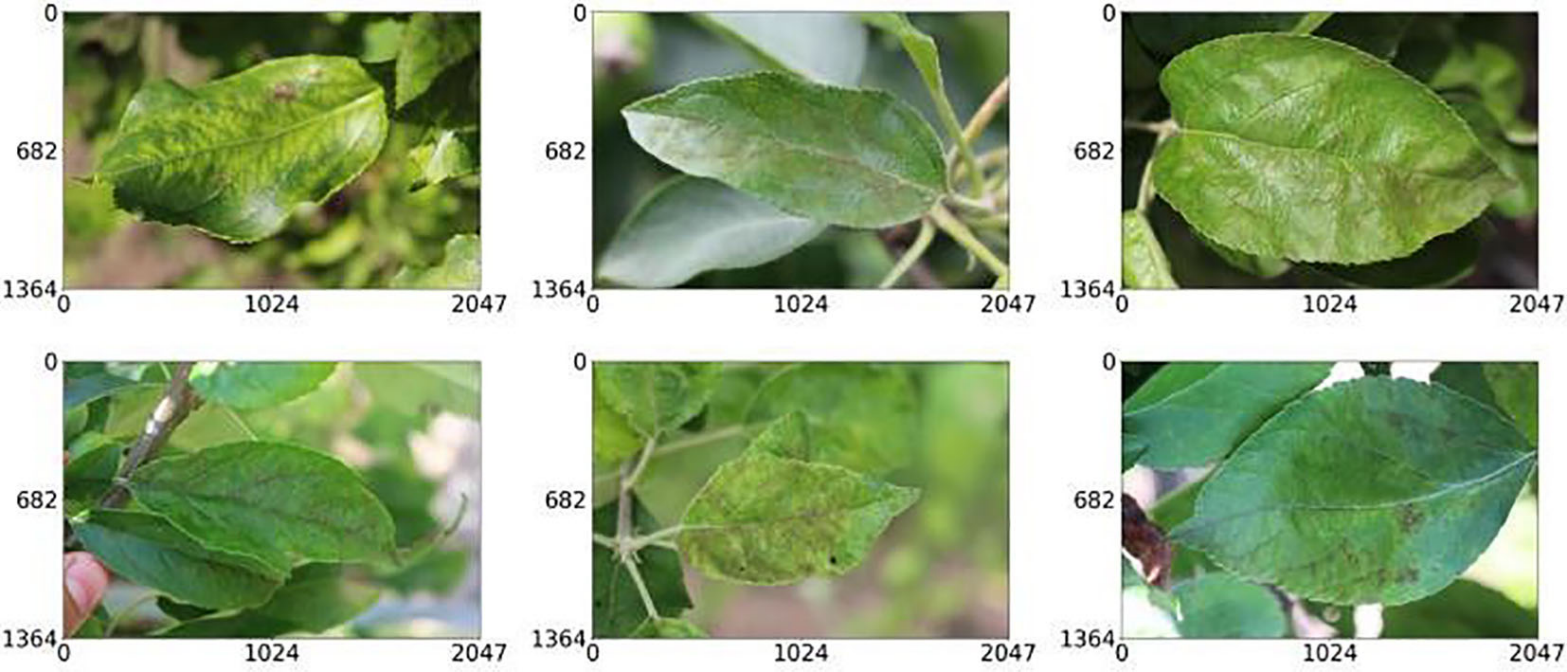
Visualization of scab leaf class.

**Figure 3:**
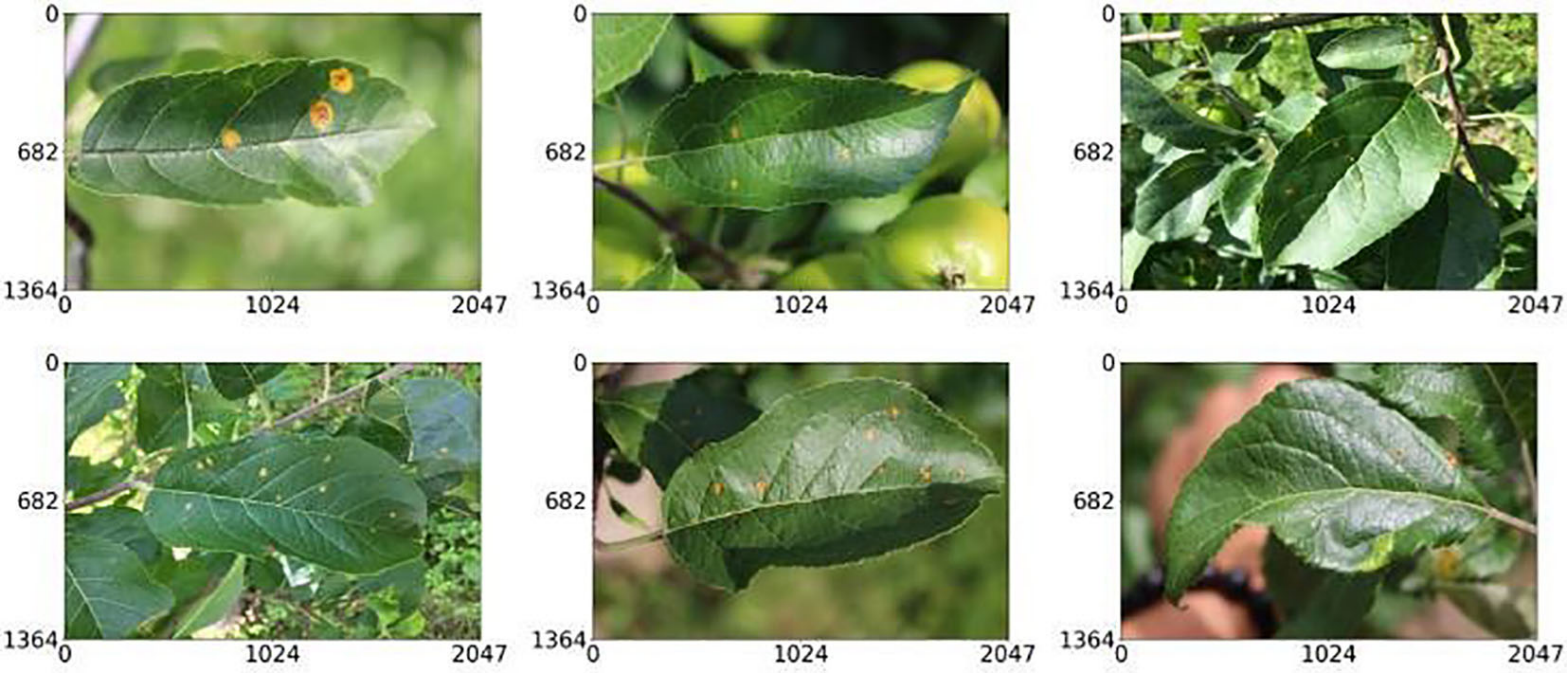
Visualization of rust leaf class.

**Figure 4:**
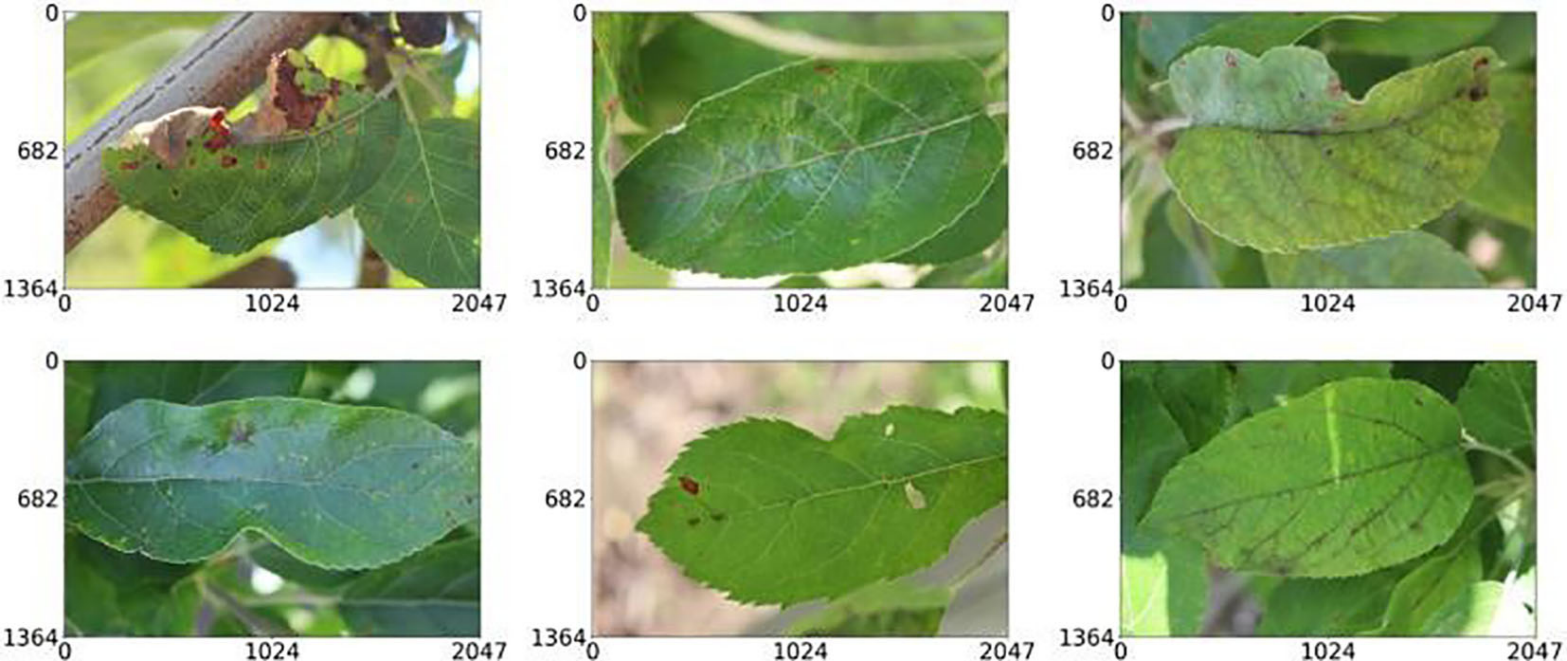
Visualization of multiple disease leaf class.

**Figures 1**, **2**, **3**, **4** presents a comparative visualization of leaf images categorized into four distinct classes: Healthy, Scab, Rust, and Multiple Disease. These representative samples serve to illustrate the visual characteristics and diversity present in each category, which is critical for model training and evaluation in plant disease classification tasks.

- The Healthy Leaf Class displays uniform coloration with no visible signs of discoloration, lesions, or deformation, providing a baseline reference for disease-free foliage.
- In contrast, the Scab Leaf Class typically exhibits dark, irregular patches or lesions concentrated in localized areas, indicative of fungal infection.
- The Rust Leaf Class is characterized by small, reddish-brown pustules or specks, often spread across the surface of the leaf, reflecting the presence of rust-causing pathogens.
- Lastly, the Multiple Disease Leaf Class includes leaves that show symptoms of more than one disease simultaneously, leading to mixed visual patterns such as overlapping spots, discoloration, and structural damage. This class introduces greater intra-class variability and poses a higher challenge for automated classification models.

### 3.2 Data pre-processing strategy

**Figure 5:**
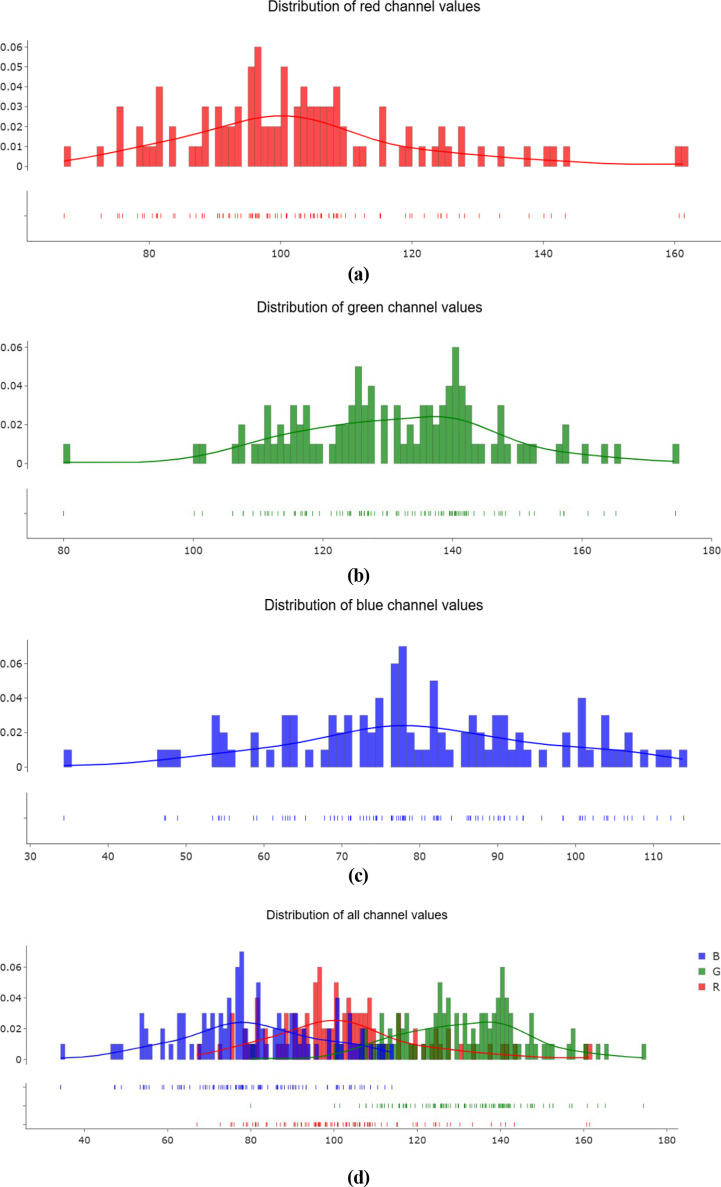
Channel distribution **(A–D).**

During dataset preparation, analyzing and standardizing the distribution of pixel intensities across image channels is a critical step to ensure consistent and effective model training. The provided study calculates the per-image mean for each RGB channel—Red, Green, and Blue—as well as the overall image brightness (**Figure 5**). This operation serves two essential purposes in the data preparation pipeline. First, it enables normalization of image data by computing the dataset’s mean per channel, which is then used to center and scale pixel values before training. Normalized inputs improve numerical stability and help neural networks converge faster and more reliably during optimization. Second, it provides a diagnostic view of the dataset, revealing potential issues such as color imbalances, exposure inconsistencies, or lighting biases, which might introduce unintended correlations or degrade model performance. For example, if the red channel consistently has higher average intensity than others, it may indicate a systematic bias (e.g., due to lighting conditions or camera sensors) that should be addressed before training.

**Figure 6:**
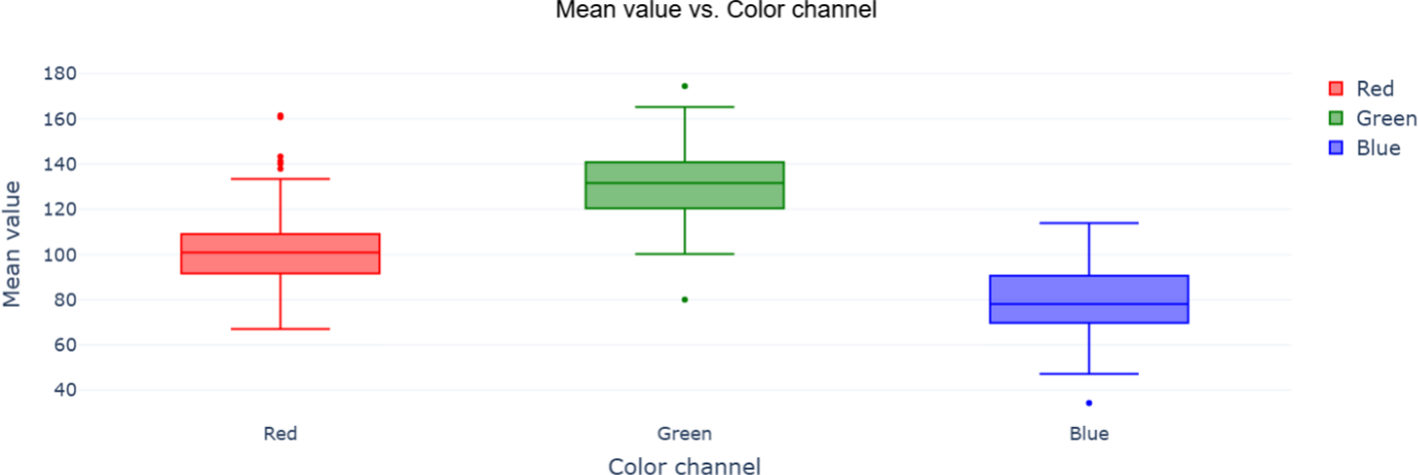
Distribution of mean pixel intensities.

**Figure 6** illustrates the distribution of mean pixel intensities across the Red, Green, and Blue (RGB) channels for all images in the dataset. This visualization plays a vital role in dataset analysis and preprocessing, particularly for image-based deep learning tasks. The **Figure 6** reveal that the Green channel exhibits the highest median mean intensity, along with the widest interquartile range, suggesting that most images are relatively brighter or more green-dominant. In contrast, the Blue channel shows the lowest median value, indicating a lower overall blue intensity in the dataset. The Red channel maintains a median around 100 but includes a greater number of outliers, suggesting a few images with unusually high red intensities—possibly due to lighting conditions or dominant red- colored objects. The disparity in channel distributions highlights a color imbalance across the dataset. Such imbalances can negatively impact model performance, particularly in convolutional neural networks, which are sensitive to variations in pixel-level statistics. To mitigate this, it is standard practice to compute and apply channel-wise normalization using the mean and standard deviation values derived from such distributions.

**Figure 7:**
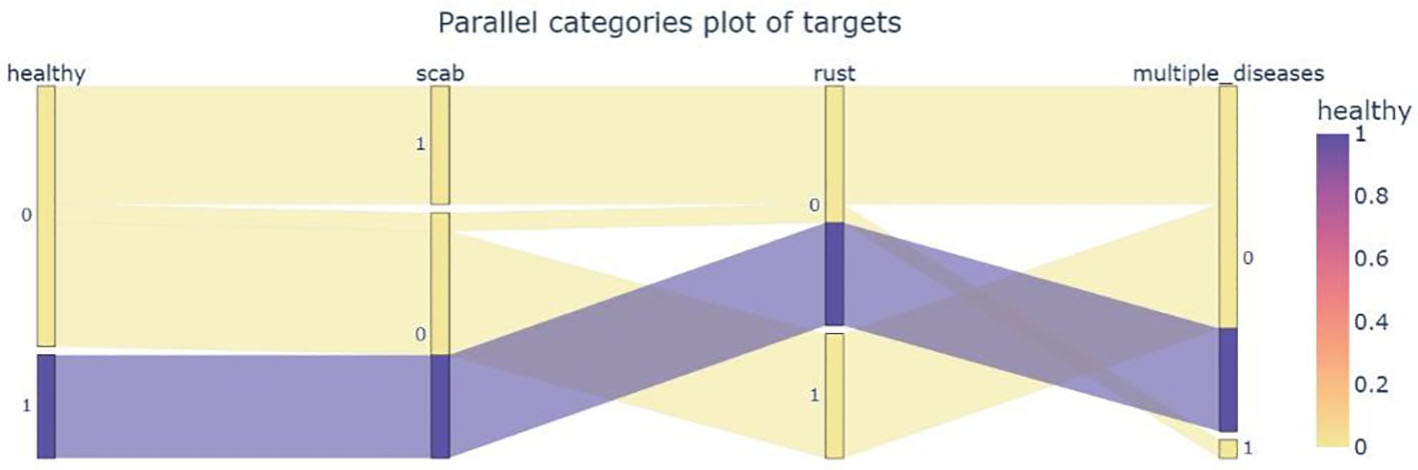
Parallel categories plot for the label distribution and co-occurrence relationships among four classes.

**Figure 7** depicts a parallel categories plot that illustrates the label distribution and co-occurrence relationships among four classes in the leaf disease dataset. Each vertical axis represents a binary classification outcome (0 or 1) for the presence or absence of a specific label, and the colored bands indicate how these labels co-occur across the dataset. The plot reveals that the dataset is predominantly composed of single-labeled samples, as most flows transition from a value of 1 on one axis to 0 on all others. For instance, a substantial portion of the data follows a path where only the healthy label is marked as present, with all disease-related labels absent. Similarly, distinct and non-overlapping flows are observed for scab and rust classes. Interestingly, the multiple diseases class shows limited co-occurrence with other classes, implying that it is treated as an independent category rather than a combination of other disease labels. The thickness of the paths also highlights class imbalance, with the healthy and rust categories appearing more frequently than scab or multiple diseases. This analysis is essential for understanding the structure of the classification problem, guiding the selection of appropriate model architectures (e.g., multiclass vs multilabel), loss functions, and preprocessing strategies such as class balancing or resampling.

**Figure 8:**
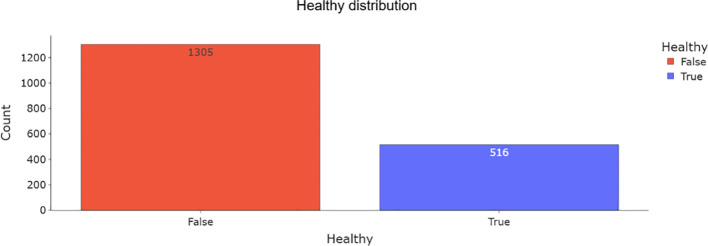
Healthy leaf (True and False) distribution.

**Figure 9:**
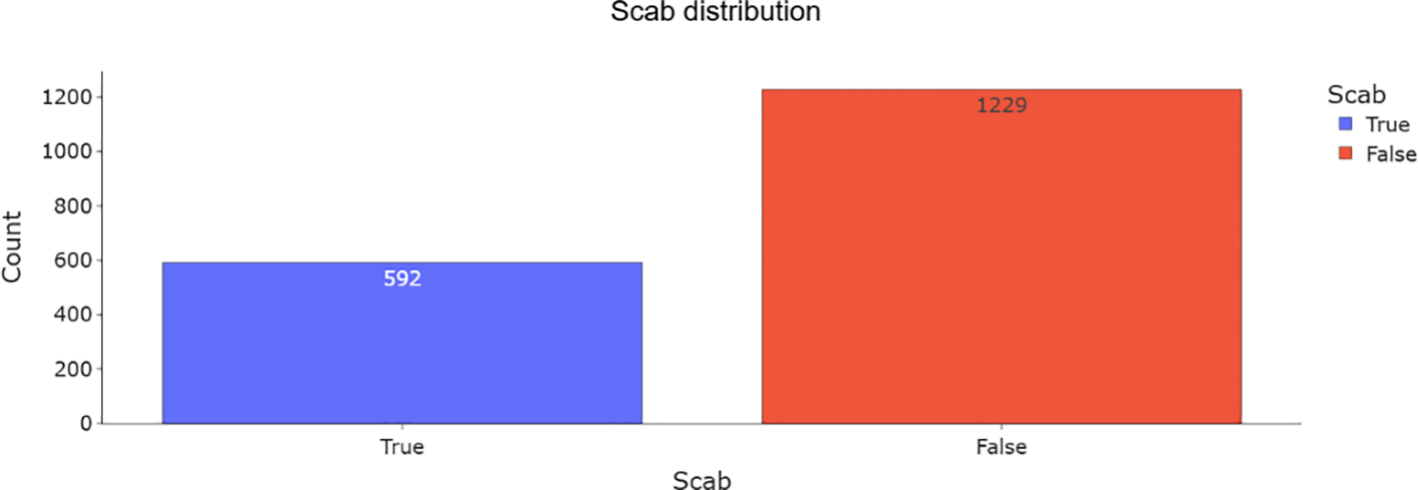
Scab leaf (True and False) distribution.

**Figure 10:**
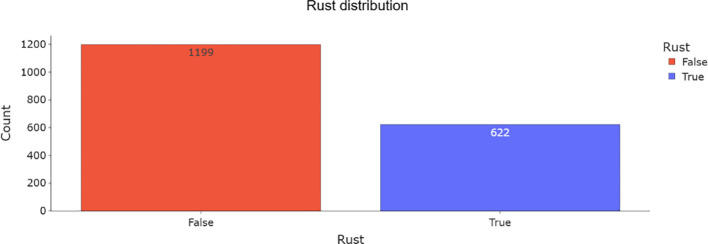
Rust leaf (True and False) distribution.

**Figure 11:**
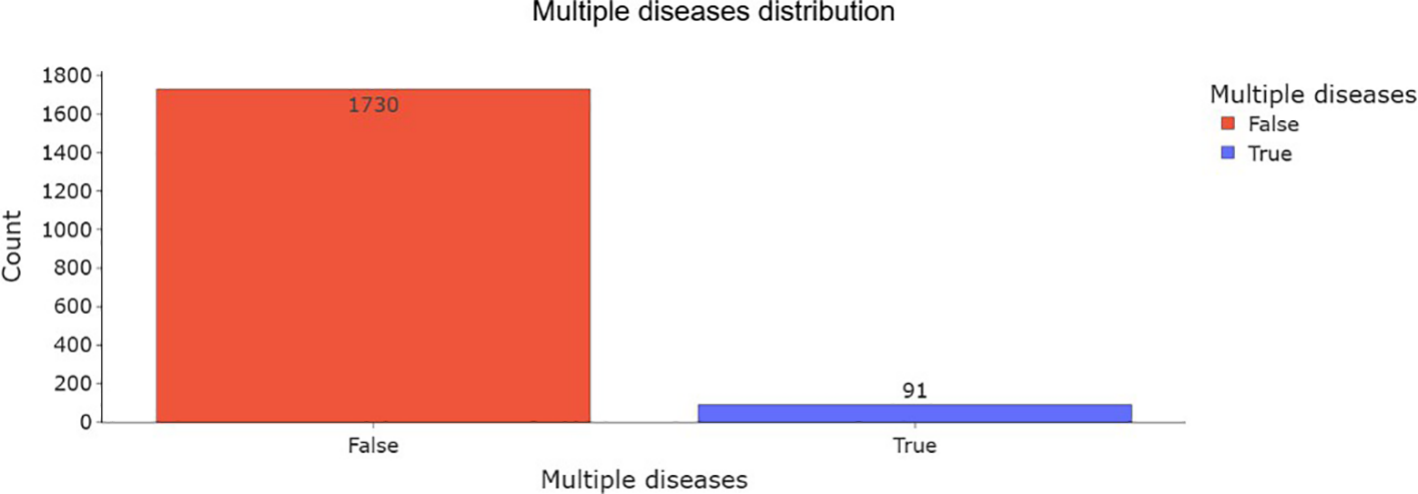
Multiple diseases (True and False) distribution.

In this study, the binary labels for healthy, scab, rust, and multiple diseases were initially transformed from numerical values to Boolean, and subsequently to string format, to facilitate categorical color mapping during visualization. This transformation allows for a clear distinction between True and False samples in each class (**Figure 8**, **9**, **10**, **11**). The resulting histogram offers an immediate and intuitive understanding of the class distribution within the dataset, which is essential for assessing model bias, ensuring training fairness, and informing the design of class reweighting strategies.

**Figure 12:**
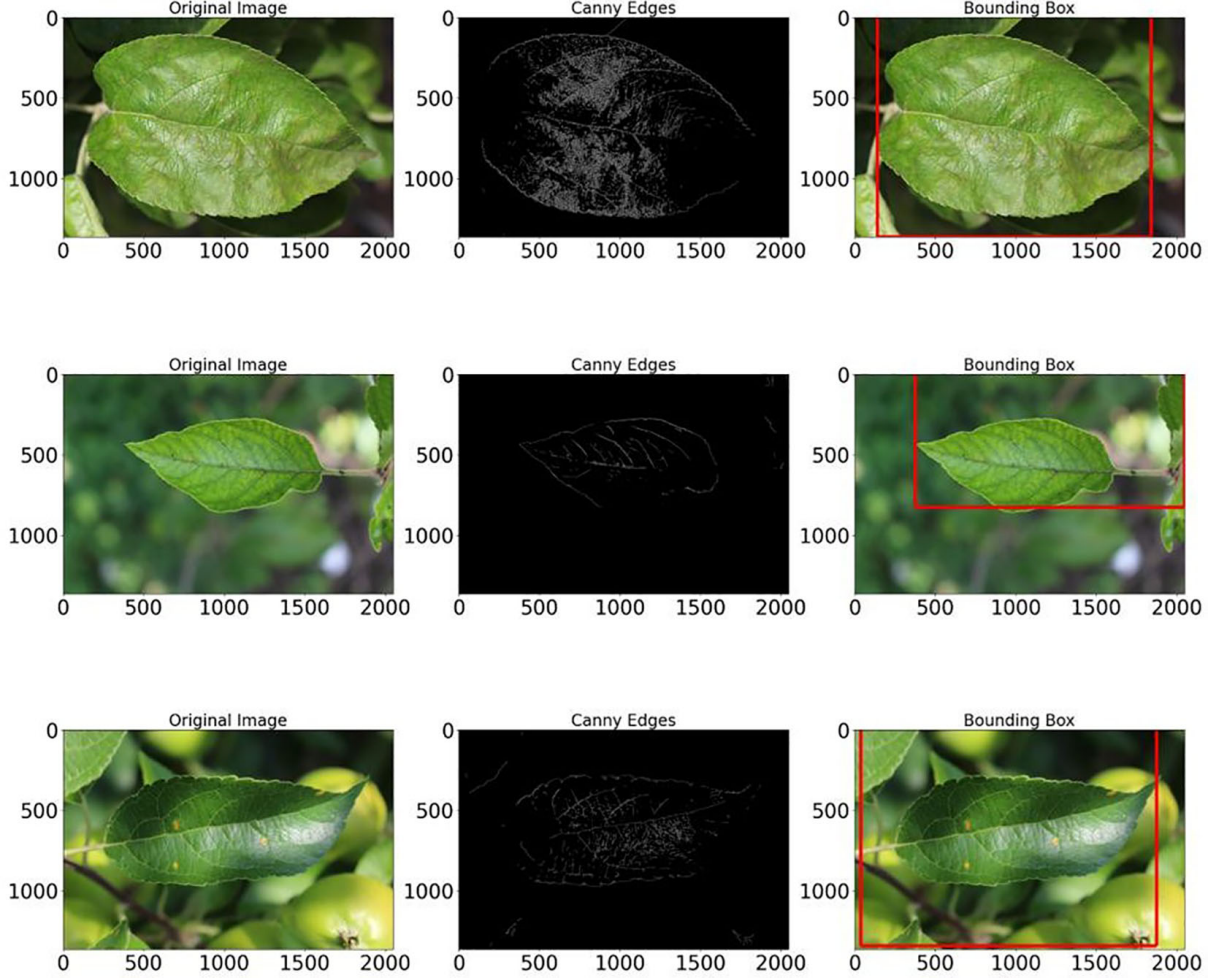
Canny Edge Detection Technique – Enhance the Structural Feature.


After performing the true–false distribution analysis to understand class balance within the dataset, the study applied the Canny edge detection technique as a subsequent preprocessing step to enhance the structural features of the input images (Medium, 2024). Canny edge detection is a robust and widely used algorithm for identifying object boundaries in images, contributing significantly to the effectiveness of image classification and segmentation models (Plant Pathology 2020 - Fgvc7; Lu Y. et al., 2022). Developed by John F. Canny in 1986, the algorithm follows a five-stage process: noise reduction, gradient computation, angle quantization, non-maximum suppression, and hysteresis thresholding. **Figure 12** unveils the output of canny edge detection technique. In the noise reduction phase, a Gaussian filter is applied to smooth the input image I(x,y), reducing high-frequency noise using the convolution:

$$I_{s}(x,y)=G(x,y)*I(x,y)$$

Where, $G(x,y)=\frac{1}{2\pi \sigma^{2}}e^{\left(-\frac{x^{2}+y^{2}}{2\sigma^{2}}\right)}$ is the Gaussian Kernal. The second step computes the gradient magnitude and direction using Sobel operators:


$$G=\sqrt{G_{x}^{2}+G_{y}^{2}},\;\theta =tan^{-1}(\frac{G_{y}}{G_{x}})$$

The angle quantization step then approximates the direction θ. In the non-maximum suppression phase, each pixel is evaluated to retain only local maxima in the direction of the gradient:


$$G(x,y)=\{\begin{array}{c} G(x,y),\;\;\;\;\;\;if\;G(x,y)>\;G_{neighbor1}\;and\;G(x,y)>\;G_{neighbor2}\\ 0,\;\;\;\;\;\;Otherwise \end{array}$$

Finally, hysteresis thresholding classifies edges using two thresholds *T_high_* and *T_low_*, retaining weak edges only if they are connected to strong ones:


$$\text{G}(\text{x},\text{y})=\{\begin{array}{c} 1,\;\;\;\;\;\;G(x,y)\geq T_{high}\\ 1,\;\;\;\;\;\;T_{low}\leq G(x,y)<T_{high}\;and\;connected\;to\;a\;strong\;edge\\ 0,\;\;\;\;\;\;Otherwise \end{array}$$

The output is a binary edge map that emphasizes object contours, which is particularly useful in plant disease detection tasks where lesion boundaries and morphological structures are crucial for classification.

In supervised learning tasks involving image-based classification, it is imperative to establish a precise and structured correspondence between raw image data and their associated categorical labels. To facilitate this, the study initially constructs absolute file paths for all training and testing samples by transforming image identifiers into fully qualified paths. This step ensures efficient and consistent access to the image data throughout the preprocessing and training pipeline.

Following path construction, the corresponding ground truth labels are extracted from the training dataset. Specifically, columns representing disease categories—commonly encoded in a one-hot or multi-label binary format, such as [‘healthy’, ‘rust’, ‘scab’]—are selected through a targeted slicing operation. These label vectors are then converted to the float32 data type, which enhances computational efficiency and maintains compatibility with widely used deep learning libraries such as TensorFlow and PyTorch.

To support rigorous model evaluation and mitigate the risk of overfitting, the dataset is further divided into training and validation subsets using a stratified random sampling approach. In this study, 15% of the data is reserved for validation, resulting in an 85:15 train-validation split. A fixed random seed (random_state=2020) is specified to guarantee reproducibility of the split across multiple experimental iterations. This stratified partitioning ensures that the distribution of class labels is preserved across both subsets, thereby enabling fair and consistent performance assessment of the classification model.

To enable efficient and scalable input handling during model training, a high-performance data pipeline is constructed using a modular data processing framework. The pipeline begins by generating a dataset that pairs image file paths with their corresponding categorical labels, ensuring that each image-label combination is treated as a unified training sample. Each image is then subjected to a decoding and preprocessing stage, which includes reading the image file from storage, converting it into a usable tensor format, resizing it to a standardized dimension, and applying normalization to standardize pixel intensity values. This processing is executed in parallel across multiple CPU threads to maximize throughput. Subsequent to decoding, the dataset undergoes a data augmentation phase, wherein controlled random transformations such as flipping, convolved, and blurring are applied. These augmentations enhance the diversity of the training set and promote generalization by exposing the model to various visual perspectives of the same class. The dataset is then configured to repeat continuously across training epochs to ensure uninterrupted data flow, and the samples are randomly shuffled to disrupt any order-based patterns, thereby reducing model bias. The data augmentation techniques are unveiled in **Table 3**.

**Table 3:** Data augmentation techniques.

Technique	Description	Mathematical formulation	Purpose in dataset
Flipping (Maharana et al., 2022; Mumuni and Mumuni, 2022)	Reverses the image along horizontal, vertical, or both axes.	Horizontal Flip: *I*′(*x*, *y*) = I(*W* − *x* − 1, *y*) Vertical Flip: *I*′(*x*, *y*) = I(*x, H* − *y* − 1)	Increases data diversity, introduces orientation invariance, and improves generalization.
Convolution (Maharana et al., 2022; Mumuni and Mumuni, 2022)	Applies filters (kernels) to extract spatial features such as edges or textures.	$I^{\prime }(x,y)=\overset{K}{\underset{i=-k}{\sum }}\overset{K}{\underset{j=-k}{\sum }}K(i,j).I(x-i,y-j)$	Enhances structural features and gradient details critical for detecting diseased patterns.
Blurring (Maharana et al., 2022; Mumuni and Mumuni, 2022)	Smooths the image to reduce noise and fine details. Typically uses Gaussian blur.	$G(x,y)=\frac{1}{2\pi \sigma^{2}}e^{-\frac{x^{2}+y^{2}}{2\sigma^{2}}}$ *I*^′^ = *G* * *I*	Simulates real-world image distortions (e.g., defocus), prevents overfitting to noise, and regularizes input.

**Figure 13:**
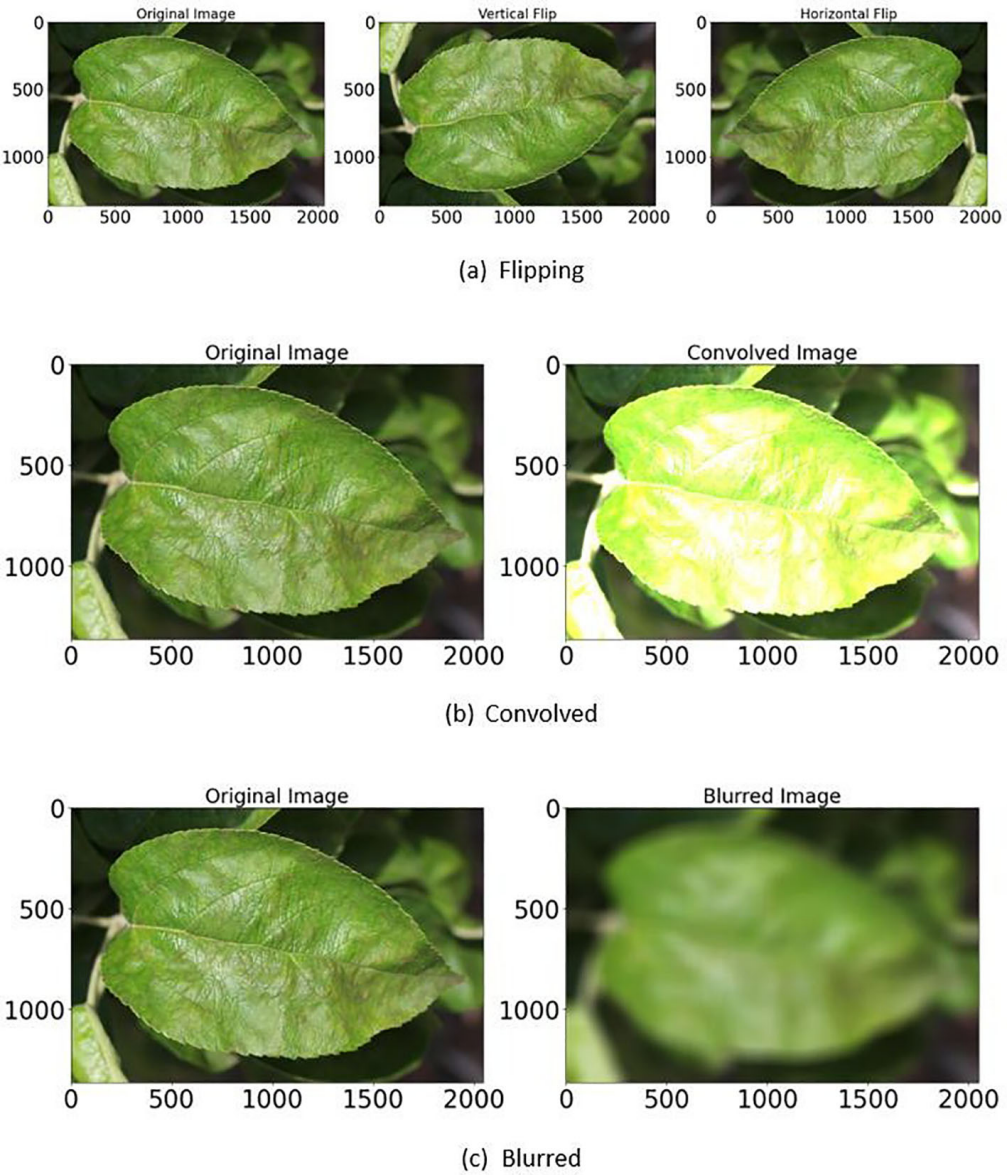
Data augmentation technique.

The output of data augmentation is shown in **Figure 13**. In order to optimize the model training process and enhance convergence, a custom learning rate schedule is implemented using a dynamic function that adjusts the learning rate across training epochs The phase wise learning rate scheduling strategy is unveiled in **Table 4**.

**Table 4:** Phase-wise learning rate scheduling strategy.

Phase	Epoch range	Learning rate behaviour	Scientific purpose
Ramp-up Phase	Epoch 0 to *ramp- up epoch*	Linearly increases from initial learning rate to a defined peak value	Prevents instability in early training by allowing gradual adaptation
Sustain Phase	Post ramp-up to *ramp-up + sustain*	Maintains learning rate at peak value	Encourages aggressive exploration in parameter space during stable learning
Exponential Decay	Remaining epochs	Exponentially decays toward a minimum threshold	Facilitates fine-tuning and convergence by reducing update magnitude
Distributed Scaling	Applied prior to training	Scales peak learning rate by the number of parallel training replicas	Ensures consistent optimization dynamics in distributed or multi- GPU environments
Scheduler Callback	Integrated during model training	Automatically adjusts the learning rate based on epoch progression	Synchronizes learning rate updates with training epochs to improve convergence

### 3.3 Methodology: integrating Squeeze-and-Excitation blocks into DenseNet for enhanced feature recalibration

**Figure 14:**
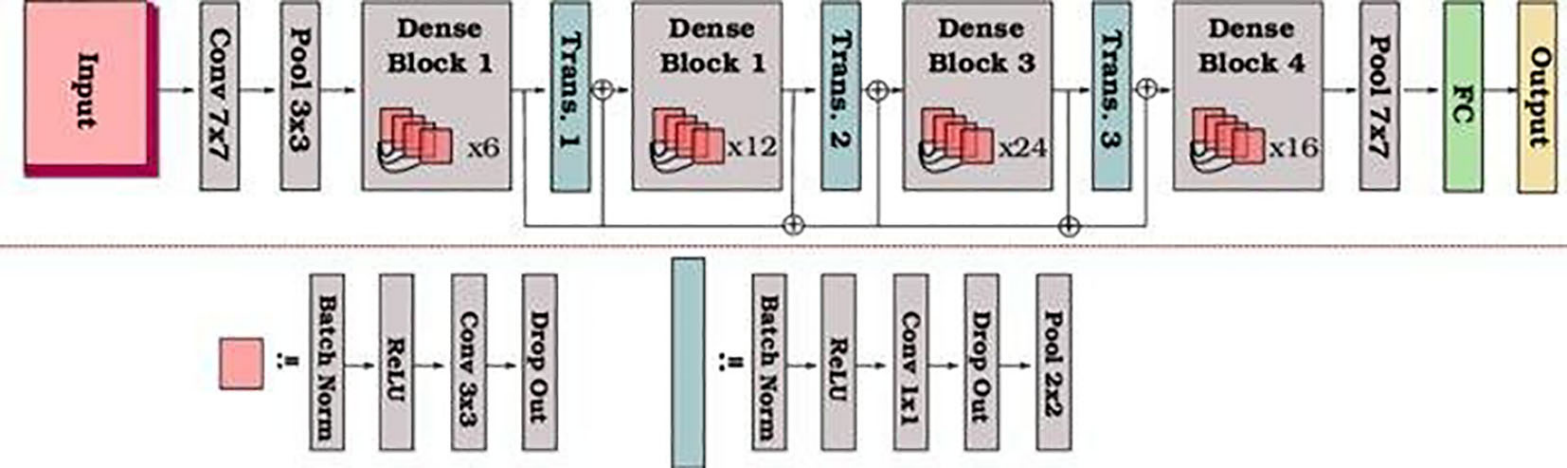
DenseNet121 Architecture as a Backbone (Maharana et al., 2022).

This methodology integrates the Squeeze-and-Excitation (SE) attention mechanism into the DenseNet121 architecture, as proposed by Jin et al. (2021). The resulting channel attention-aware network dynamically recalibrates feature maps by learning which channels contain the most informative features, as shown in **Figure 14**. **Table 5** unveils the summary architecture of DenseNet121.

**Table 5:** DenseNet121 summary architecture.

Stage	Layer type	Output shape	Details
Input	Input Layer	512×512×3	RGB image
Conv1	7×7 Conv, stride 2	256×256×64	$\text{Output:}[\frac{512-7+2\cdot 3}{2}+1]=256$
	3×3 Max Pool, stride 2	128×128×64	Downsample
Dense Block 1	6 Dense Layers	128×128×256	Growth Rate = 32 → 6×32 + 64 = 256 channels
Transition 1	1×1 Conv + 2×2 AvgPool, stride 2	64×64×128	Compression θ = 0.5
Dense Block 2	12 Dense Layers	64×64×512	12×32 + 128 = 512
Transition 2	1×1 Conv + 2×2 AvgPool, stride 2	32×32×256	θ = 0.5
Dense Block 3	24 Dense Layers	32×32×1024	24×32 + 256 = 1024
Transition 3	1×1 Conv + 2×2 AvgPool, stride 2	16×16×512	θ = 0.5
Dense Block 4	16 Dense Layers	16×16×1024	16×32 + 512 = 1024
Final Layers	Global Average Pooling	1×1×1024	GAP over 16×16 spatial size
	Fully Connected (Dense Layer)	1×1×C	C = number of classes (e.g., 10, 100, etc.)
	Softmax	1×1×C	Classification probabilities


$${\hat{y}}_{y}=\frac{\exp(w_{j}^{T}z+b_{j})}{\sum_{k=1}^{K}exp(w_{k}^{T}z+b_{k})}$$

The framework consists of two major components:

- DenseNet Backbone: Efficient and compact feature extraction through dense connections.
- Squeeze-and-Excitation Blocks: Improve channel-wise feature representation by modelling interdependencies between feature channels.

#### 3.3.1 DenseNet121 architecture

DenseNet121 is part of the Densely Connected Convolutional Networks (DenseNet) family proposed by Huang et al., 2017 (Ahmed, 2021). The core innovation lies in its dense connectivity pattern, where each layer is connected to every other layer in a feed-forward fashion.

Let’s denote the output of the *l^th^* layer as *x_l_*. Then, instead of computing *x_l_* from just *x_l_*−1, DenseNet computes:


$$x_{l}=H_{l}([x0,x1,x2,x3,\ldots \ldots ,x_{l-1}])$$

Where

[*x*_0_, *x*_1_, *x*_2_, *x*_3_…, *x*_l_-1 denotes concatenation of feature maps produced by all preceding layers,

• *H*_l_(.) is a composite function including Batch Normalization (BN), Rectified Linear Unit (ReLU), and convolution (Conv).

DenseNet121 architectural components is composed of:

• Dense blocks, each with multiple composite layers: Each dense block contains a number of densely connected dense layers, where each layer appends *k* new feature maps to the input feature map. This *k* is called the growth rate.

Let:

o *x*_0_ ∈ R^H×W×C_0_^: Input to the block

o L: Number of layers in the block

Then, the output of the dense block is:


$$x_{out}=[x0,x1,x2,x3,\ldots \ldots ,x_{l-1}]\in R^{H\times W\times (C_{0}+L\cdot k)}$$

This linear growth of feature maps is far more efficient than traditional deep networks. To mitigate computational cost, DenseNet uses bottleneck layers, which consist of:

o 1×1 convolution to reduce feature dimensions
o Followed by a 3×3 convolution

This reduces the number of input channels to the expensive 3×3 convolution operation.

**•** Transition layers between dense blocks to downsample the feature maps

Between dense blocks, DenseNet uses transition layers to:

o Reduce the number of feature maps

o Perform downsampling (spatial resolution reduction) This is done via:

o 1×1 convolution (for feature compression)

o 2×2 average pooling (for spatial downsampling)

Let *C* be the number of input channels, and θ ∈ (0,1] the compression factor, then the number of output channels is:

*C*_out_ = [θ · *C*]

Typically, θ = 0.5, halving the number of channels and improving efficiency.

**•** A global average pooling and fully connected (softmax) layer at the end

At the end of the final dense block, DenseNet121 applies Global Average Pooling (GAP) to produce a vector of size *C*:


$$z_{i}=\frac{1}{H.W}\overset{H}{\underset{h=1}{\sum }}\overset{W}{\underset{W=1}{\sum }}x_{i},\;h,\;w,\;\;\;i=1,\ldots .,C$$

Then a fully connected layer followed by softmax activation is applied for classification:


$${\hat{y}}_{y}=\frac{\exp(w_{j}^{T}z+b_{j})}{\sum_{k=1}^{K}exp(w_{k}^{T}z+b_{k})}$$

Where

o $\hat{y}$_y_: Probability of class *j*
o *z*: Feature vector from GAP
o K: Number of classes
o *w_j_*, *b_j_*: Weights and bias for class *j*

#### 3.3.2 Squeeze-and-Excitation blocks

In traditional CNNs, convolutions operate locally in the spatial domain (height and width), aggregating information from neighboring pixels via kernels. However, all channels in a convolutional output tensor are treated equally. CNNs lack mechanisms to dynamically prioritize or suppress feature maps (channels) based on their relevance to a given task.

This is where the SE block comes in—it enables adaptive recalibration of channel-wise features by learning inter- channel dependencies. Instead of assuming all channels contribute equally, the SE block learns attention weights for each channel and modulates the feature maps accordingly (Ahmed, 2021; C. R et al., 2023). The SE block learns to scale each channel of a feature map with a learned weight based on the global context of the image (Samavati, 2023). This allows the network to focus on the most informative features, suppressing irrelevant ones.

The SE block is composed of three sequential operations:

• Squeeze – global spatial information is compressed into a channel descriptor.
• Excitation – inter-channel relationships are captured through a gating mechanism.
• Scale – input channels are modulated based on learned importance weights. Let’s denote the input feature map as:

$$U\in R^{H\times W\times C}$$

Where:

• *H*, *W* = spatial dimensions
• *C* = number of channels

### Step 1: Squeeze — global information embedding

In this part performs Global Average Pooling (GAP) to generate a vector *z* ∈ *R^c^*, summarizing the spatial information for each channel:


$$z_{c}=\frac{1}{H.W}\sum_{i=1}^{H}\sum_{j=1}^{W}U_{C}(i,j)$$

This creates a channel descriptor that encodes global contextual information for each feature channel.

### Step 2: Excitation — adaptive channel reweighting

We pass the vector *z* through a bottleneck of two fully connected (FC) layers to capture non-linear channel dependencies:


$$s=\sigma (W_{2}\cdot \delta (W_{1}\cdot z))$$

Where

• *W*1 ∈ $R^{\frac{C}{r}\times C}$: reduction (bottleneck) FC layer $W_{2}\in R^{c\times \frac{C}{r}}\;\;\;\;$expansion FC layer
• *δ*: ReLU activation function
• *σ*: Sigmoid activation function (for attention gating)
• *r*: Reduction ratio (e.g., 16)

This process allows the block to learn which channels to emphasize or suppress based on the global context.

### Step 3: Scale — feature recalibration

Finally, each channel of the original feature map *U_c_*; is scaled by the learned weight *s_c_*:


$$U_{c}=s_{c}\cdot U_{c}$$

The output *U* ∈ *R*^*H*×*W*×*C*^ is a channel-wise modulated version of the input, with:

• Important features amplified
• Less useful features diminished

## 4 Measurement parameter

In a plant disease classification problem involving four classes — healthy, scab, rust, and multiple_diseases — a neural network outputs a probability distribution across these categories for each input image. The true label is represented using a one-hot encoded vector, where only the correct class is marked with a 1 and the rest with 0s. For example, if an image actually shows rust, its true label vector would be (0, 0, 1, 0). To evaluate how well the model’s prediction aligns with the true label, we use the categorical crossentropy loss function. This function measures the distance between the predicted probability distribution and the true distribution (i.e., the one-hot encoded vector). Mathematically, the loss for a single sample is calculated using the formula:


$$Loss=-\sum_{i=0}^{k-1}y_{i}\cdot log({\hat{y}}_{i})$$

Here, *y_i_* is the true label for class i and *y_i_* is the predicted probability for class i. Since only one *y_i_* is 1, the equation simplifies to taking the negative logarithm of the predicted probability for that true class:

$Loss=-\log({\hat{y}}_{true\;class})$*Loss* = -log (*y*_trueclass_)

This means that if the model assigns a high probability to the correct class, the loss is small — indicating a good prediction. Conversely, if the probability is low, the loss increases sharply, penalizing the model for poor confidence in the right class. When training on a batch of data, the average loss across all samples is computed using the batch-wise formula:


$$Loss=-\frac{1}{N}\sum_{n=1}^{N}\sum_{i=0}^{K-1}y_{n,i}.log(y_{n,i})$$

This version helps the model adjust its parameters using gradients computed from multiple examples at once. It’s important to note that categorical crossentropy requires the labels to be one-hot encoded. If instead the labels are provided as integers (e.g., 2 for rust), the appropriate version to use is sparse categorical crossentropy, which internally converts the integer labels to one-hot format during loss computation.

While the loss function tells us how wrong the model is, an optimizer tells us how to adjust the model to be less wrong. Adam, short for Adaptive Moment Estimation, is one of the most popular and efficient optimization algorithms used in training deep learning models.

Adam works by calculating gradients (direction and rate of change) for each parameter and then using two ideas to improve learning: momentum, which helps smooth out the updates using past gradients, and adaptive learning rates, which let each parameter have its own learning rate that adjusts over time. This combination makes Adam fast, reliable, and robust, even in problems with noisy or sparse gradients (like NLP tasks). The **Table 6** and **7** unveil the key points and evaluation metrices, respectively.

**Table 6:** Key point.

Feature	Categorical crossentropy	Adam optimizer
Purpose	Measures prediction error (loss)	Adjusts model parameters to minimize loss
Type	Loss function	Optimization algorithm
Used In	Multi-class classification tasks	Training of neural networks
Input Format	One-hot encoded labels and probability predictions	Gradient values of loss w.r.t. weights
Mathematical Expression	*y_i_ log*( $\hat{y}$*_i_*)	Combines momentum + RMSProp formulas
Key Parameters	No parameters (but requires correct label format)	learning_rate, beta_1, beta_2, epsilon
When to Use	When labels are one-hot encoded and multiple classes exist	For most deep learning models, especially where adaptive learning is helpful
Common Mistake	Using it with non-one-hot labels (use sparse version instead)	Ignoring learning rate tuning for complex datasets

**Table 7:** Evaluation Matrices.

Metric	Definition	Formula	Role/what it measures	Interpretation	Strengths	Weaknesses	Real-world example
Accuracy	Proportion of total predictions that are correct	$\frac{TP+TN}{TP+TN+FP+FN}$	Overall correctness	Out of all predictions, how many were right?	Simple and intuitive; useful for balanced datasets	Misleading in imbalanced data	Classifying emails into spam/not spam where spam and non- spam are equally common
Precision	Proportion of predicted positives that are actually correct	$\frac{TP}{TP+FP}$	Positive prediction confidence	When the model says “positive,” how often is it correct?	Good for minimizing false alarms	Can miss many real positives if overly cautious	Email spam filter: ensures genuine emails aren’t marked as spam
Recall	Proportion of actual positives that were correctly predicted	$\frac{TP}{TP+FN}$	Sensitivity or coverage of positives	Out of all actual positives, how many did the model find?	Good at capturing all relevant cases	May increase false positives if too aggressive	Medical diagnosis: ensure no cancer case is missed
F1 Score	Harmonic mean of precision and recall	$\frac{2TP}{2TP+FP+FN}$	Balanced measure of correctness and completeness	How well does the model balance false positives and false negatives?	Penalizes extreme values of precision/recall; balanced metric	Hard to interpret alone; doesn’t distinguish between types of errors	Information retrieval: search engine results need to be relevant and exhaustive

## 5 Result

The **Table 8** presents the performance metrics of the AgriX-SENet model for both the training and validation datasets, evaluated using average accuracy and average loss.

**Table 8:** Performance metrices of the AgriX-SENet.

S no	Model	Training		Validation	
		Average accuracy	Average loss	Average accuracy	Average loss
1	**AgriX-SENet**	0.9747	0.0822	0.9507	0.1590

For the training phase, AgriX-SENet achieved a high average accuracy of 97.47% with a low average loss of 0.0822, indicating that the model effectively learned the patterns in the training data with minimal error. During validation, the model maintained a strong performance with an average accuracy of 95.07%, accompanied by a slightly higher average loss of 0.1590. This slight increase in loss and marginal drop in accuracy on the validation set is expected and reflects a normal generalization behavior without signs of overfitting. The close alignment between training and validation metrics confirms that AgriX-SENet is both accurate and robust, with good generalization capabilities when applied to unseen data.

**Figure 15:**
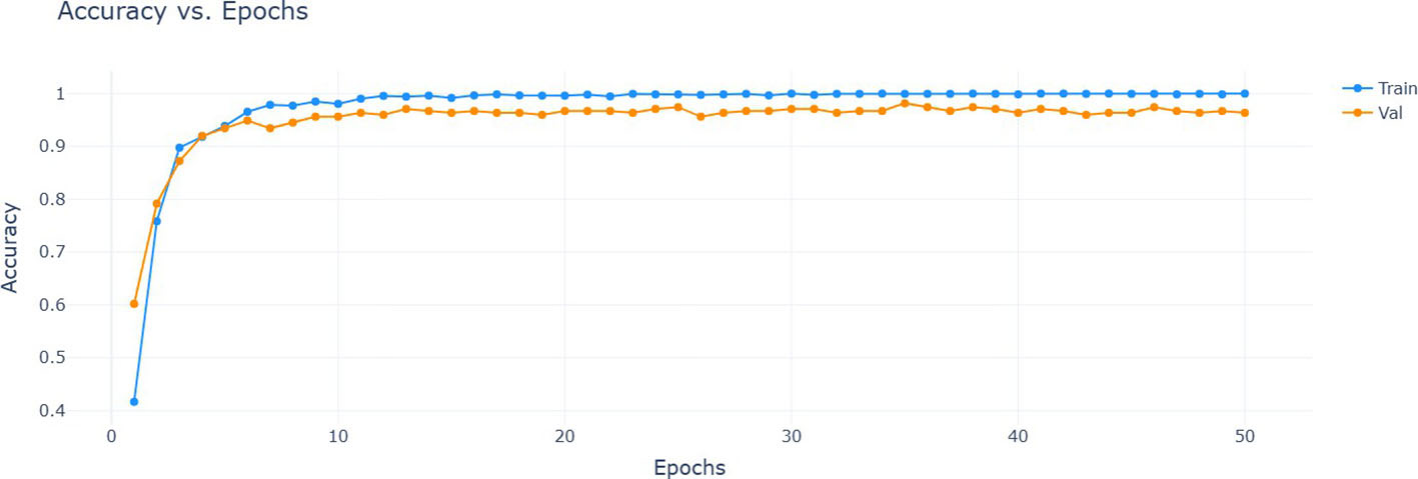
Accuracy vs. Epochs.

The accuracy vs. epochs graph (**Figure 15**) illustrates the performance of the model over 50 training epochs for both training and validation datasets. During the initial epochs, both training and validation accuracies increase rapidly, indicating effective learning and convergence. The training accuracy continues to improve steadily, eventually stabilizing near 1.0000, while the validation accuracy also plateaus at a high level around.9600. The close alignment between training and validation accuracies over the remaining epochs suggests that the model is learning robust features and generalizing well to unseen data. The stability of the validation accuracy across epochs further confirms that the model maintains consistent performance without significant degradation.

**Figure 16:**
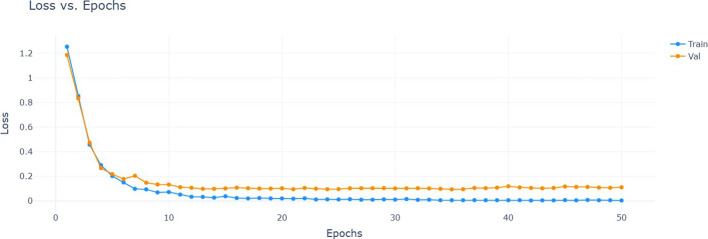
Loss vs Epochs.

The loss vs. epochs graph (**Figure 16**) provides insight into the model’s convergence behavior during training and validation over 50 epochs. Initially, both training and validation losses decrease rapidly, indicating effective learning and a strong reduction in prediction error. After approximately 10 epochs, the training loss continues to decrease gradually and stabilizes near zero, reflecting a high degree of fit to the training data. Similarly, the validation loss decreases significantly in the early stages and then levels off at a low value, demonstrating that the model generalizes well to unseen data. The close alignment and consistent behavior of the training and validation loss curves throughout the training process suggest that the model is well-optimized and does not suffer from overfitting or underfitting.

The classification report for the AgriX-SENet model provides a detailed evaluation of its performance across four disease categories using key metrics: precision, recall, and F1 score (shown in **Table 9**).

**Table 9:** Classification report.

S No	Model	Parameter	Precision	Recall	F1 score
1	**AgriX-SENet**	healthy	0.97	1.00	0.99
		scub	1.00	0.38	0.56
		rust	0.99	1.00	0.99
		multiple diseases	0.93	0.98	0.95
		macro average	0.97	0.84	0.87
		weighted average	0.97	0.96	0.96

• For the “healthy” class, the model achieved a precision of 0.97, a recall of 1.00, and an F1 score of 0.99, indicating that nearly all healthy samples were correctly identified with very few false positives.
• In the case of “scab”, the model showed perfect precision (1.00), meaning every prediction of scab was correct. However, the recall was low at 0.38, suggesting that the model missed many actual scab cases. This led to a lower F1 score of 0.56, indicating a trade-off between precision and recall.
• For the “rust” class, both precision and recall were extremely high (0.99 and 1.00, respectively), resulting in an F1 score of 0.99, demonstrating excellent classification performance.
• The “multiple diseases” class also showed strong performance, with precision of 0.93, recall of 0.98, and an F1 score of 0.95, reflecting the model’s ability to accurately detect this complex class.

The macro average (which treats all classes equally) shows a precision of 0.97, recall of 0.84, and F1 score of 0.87, reflecting the imbalance caused mainly by the low recall in the scab class. Meanwhile, the weighted average (which considers class support) indicates more balanced performance with precision, recall, and F1 score all around 0.96–0.97, showing that the model performs well overall despite some weaknesses in specific classes. This analysis suggests that while AgriX-SENet is highly effective in most categories, further attention may be needed to improve recall for the scab class.

**Figure 17:**
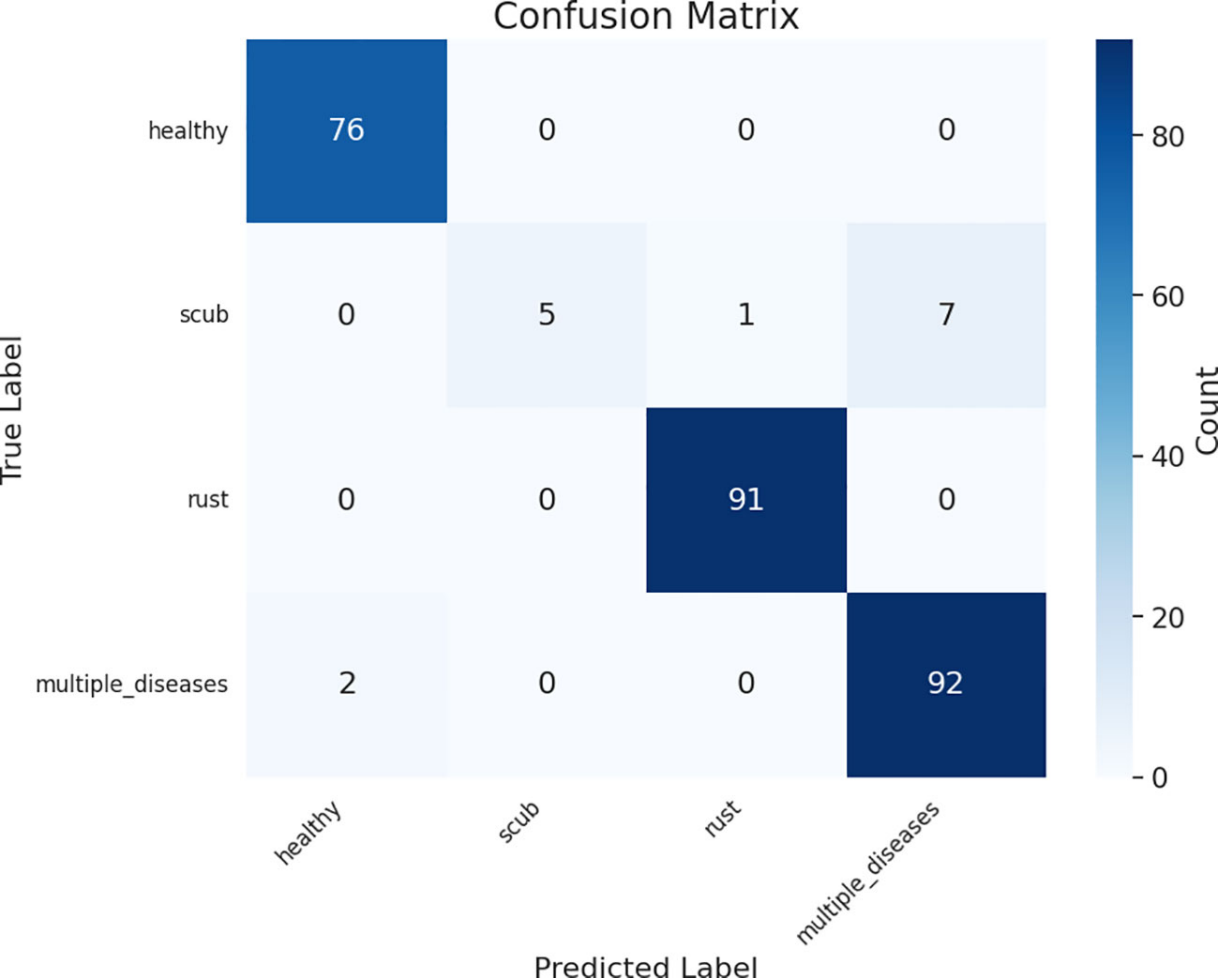
Confusion matrix.

The confusion matrix (**Figure 17**) offers a detailed qualitative assessment of the AgriX-SENet model’s classification capabilities across four distinct categories: healthy, scab, rust, and multiple diseases. The model exhibits strong performance in correctly identifying healthy and rust categories, with minimal to no observable misclassification. Additionally, it demonstrates high reliability in recognizing the multiple diseases class, with only occasional confusion with the healthy class, indicating robust generalization to complex pathological conditions.

However, the scab category presents a noticeable challenge. The model frequently misclassifies scab samples, particularly as multiple diseases or rust. This misclassification suggests difficulty in distinguishing scab-specific features, possibly due to visual similarities with other disease symptoms or limited representation during training.

This observation aligns with performance limitations highlighted in the corresponding classification metrics and suggests an area for targeted improvement.

**Figure 18:**
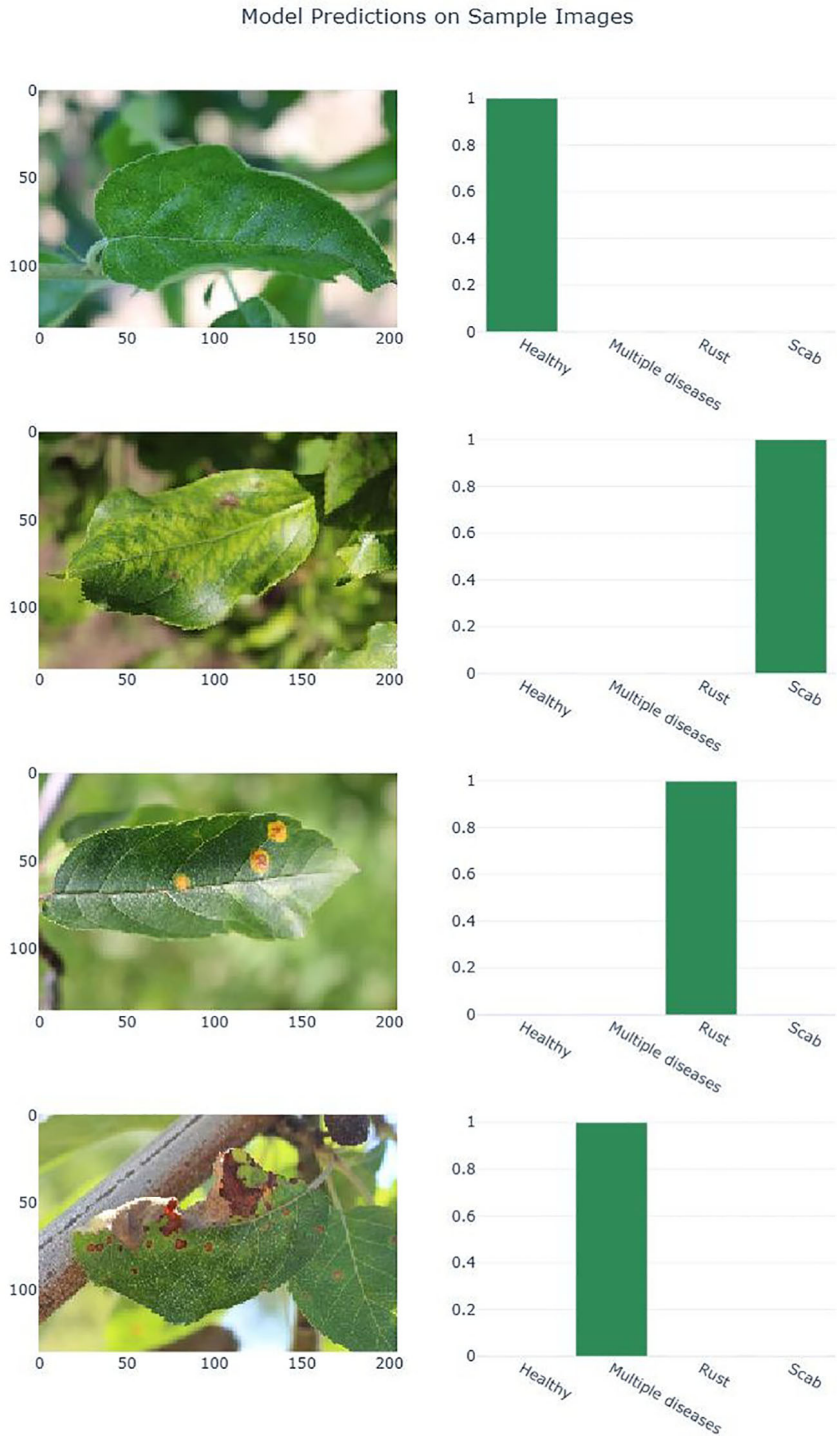
AgriX-SENet model’s predictions.

**Figure 18** presents qualitative results showcasing the AgriX-SENet model’s predictions on a set of representative leaf samples, each associated with one of the target classes: healthy, scab, rust, and multiple diseases. For each input image, the corresponding bar graph illustrates the model’s confidence scores across all classes. The visual analysis confirms the model’s high discriminative capability, as it assigns a dominant prediction score to the correct class in each instance. Healthy leaf samples are confidently classified as healthy, reflecting the model’s ability to distinguish uninfected foliage. Similarly, disease-specific features such as necrotic spots and rust pustules are effectively captured, enabling accurate classification of scab and rust conditions.

Notably, the model also demonstrates robustness in handling visually complex cases, such as multiple diseases, where symptoms of more than one infection co-exist. The associated prediction in such cases remains decisive, indicating that the model can generalize well across varying patterns of pathology. These visualizations offer intuitive evidence of the model’s interpretability and its ability to maintain high confidence in its predictions across diverse leaf conditions.

To enhance the interpretability of the AgriX-SENet model, Gradient-weighted Class Activation Mapping (Grad- CAM) was utilized to generate class-discriminative localization maps. These visualizations highlight the specific regions within the input image that the model focuses on while making a classification decision, thus providing insight into its internal reasoning.

**Figure 19:**
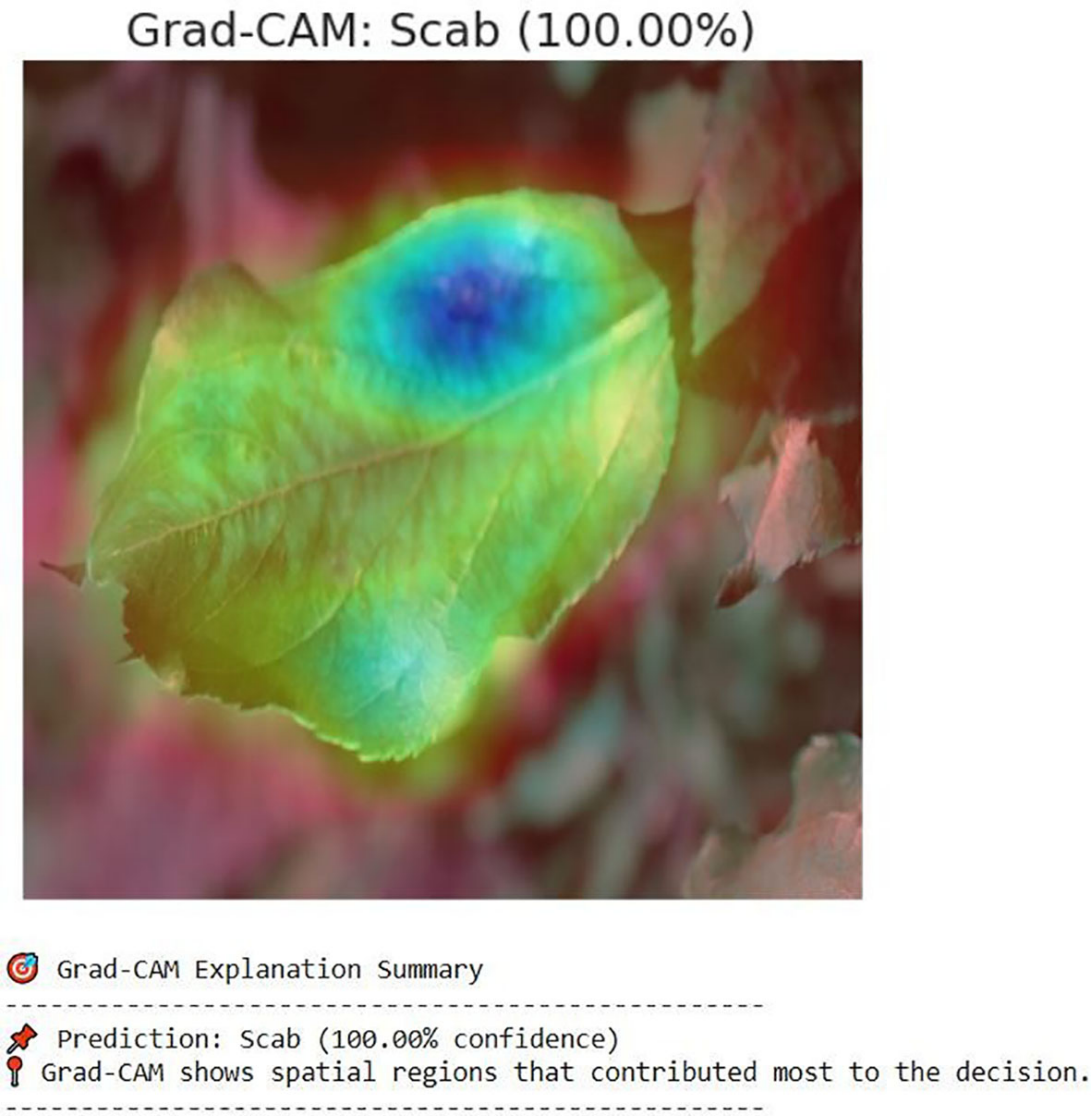
Grad-CAM explanation.

**Figure 19** illustrates an example Grad-CAM overlay for a sample classified as scab. The model’s prediction is accompanied by a high level of confidence, and the heatmap reveals that the most influential regions correspond to the symptomatic lesion areas on the leaf surface. Warmer regions (in red) indicate higher activation, suggesting that the network strongly associates these areas with the scab class.

The Grad-CAM implementation was carried out through a dedicated pipeline. The input image was first resized and normalized using the preprocess to ensure compatibility with the model’s input shape and dynamic range. Then, gradcam heatmap computed the gradients of the target class with respect to the activations in a chosen convolutional layer. These gradients were globally averaged to derive a weight for each channel, which was then used to generate a coarse heatmap that reflects the spatial importance of each pixel in the classification.

Finally, the end-to-end visualization process by overlaying the heatmap on the original image. This helped validate that the model’s predictions were based on relevant pathological patterns rather than unrelated background features. Such visual explanations are critical in building trust in deep learning systems deployed in real-world agricultural settings, where interpretability can aid agronomists and farmers in validating disease detection outcomes.

To further enhance the interpretability and transparency of the proposed AgriX-SENet model, SHapley Additive exPlanations (SHAP) were employed to analyze the model’s predictions at a feature level. While Grad-CAM provides spatial insight into which regions in the image influenced the decision, SHAP offers a feature-level breakdown of how much each extracted feature contributes—positively or negatively—to the final classification outcome.

**Figure 20:**
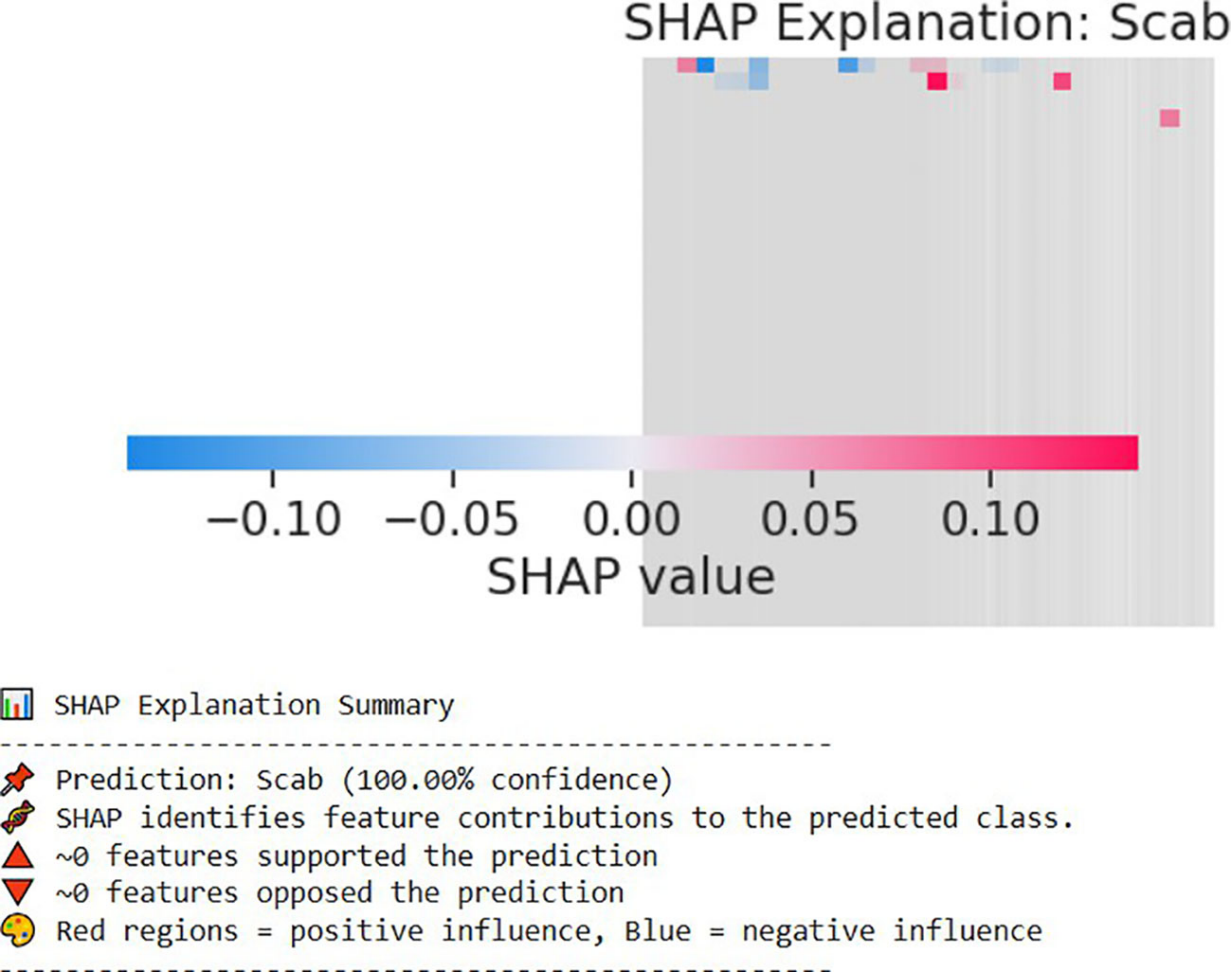
SHAP explanation.

**Figure 20** illustrates the SHAP explanation for a leaf sample classified as scab. The model yielded a high- confidence prediction, and the SHAP value plot decomposes the decision into additive feature contributions. Red- colored bars denote features that supported the prediction (positive SHAP values), while blue bars indicate features that contradicted it (negative SHAP values). The relative SHAP value magnitudes reflect the strength of influence each feature had on the model’s decision boundary.

The underlying methodology, is summarized below:

- Image Preprocessing: The selected image is resized and normalized via preprocess_image() to ensure consistent input format. This processed tensor is then passed through the model for prediction.
- Feature Extraction: To explain the model’s decision in terms of abstract features rather than raw pixels, a truncated model was constructed using the layers preceding the final dense classifier. A GlobalAveragePooling2D layer was used to obtain a flattened feature vector.
- SHAP Setup: A lightweight replica of the original classification head (softmax layer) was reconstructed using Keras. This head was used in conjunction with the extracted feature vector to estimate the SHAP values via shap.KernelExplainer. A small set of background feature vectors was drawn from the training set to represent the data distribution.
- SHAP Explanation Generation: The SHAP explainer computed feature attributions for the selected test sample. Each value indicated whether a particular feature increased or decreased the likelihood of the predicted class. The values were then visualized as a SHAP summary plot showing a distribution of influences.

This approach offers a complementary explanation to the Grad-CAM heatmap, allowing for a more granular understanding of the model’s behavior. In this specific case, the SHAP plot reveals that a majority of the feature activations aligned with the diagnosis of scab, further validating the robustness and reliability of the model.

In the LIME visualizations, positively contributing regions (green superpixels) generally correspond to disease-affected portions of the leaf, including lesions, discoloration, necrotic areas, and other pathological characteristics that support the predicted class. In contrast, negatively contributing regions (red superpixels) typically represent healthy leaf tissue, visually neutral regions, or background areas that do not contain discriminative disease features. Consequently, these regions either contribute little to the classification decision or provide evidence against the predicted disease category. The spatial distribution of positive and negative superpixels further confirms that the model primarily relies on biologically meaningful disease symptoms rather than irrelevant background information when generating predictions.

**Figure 21:**
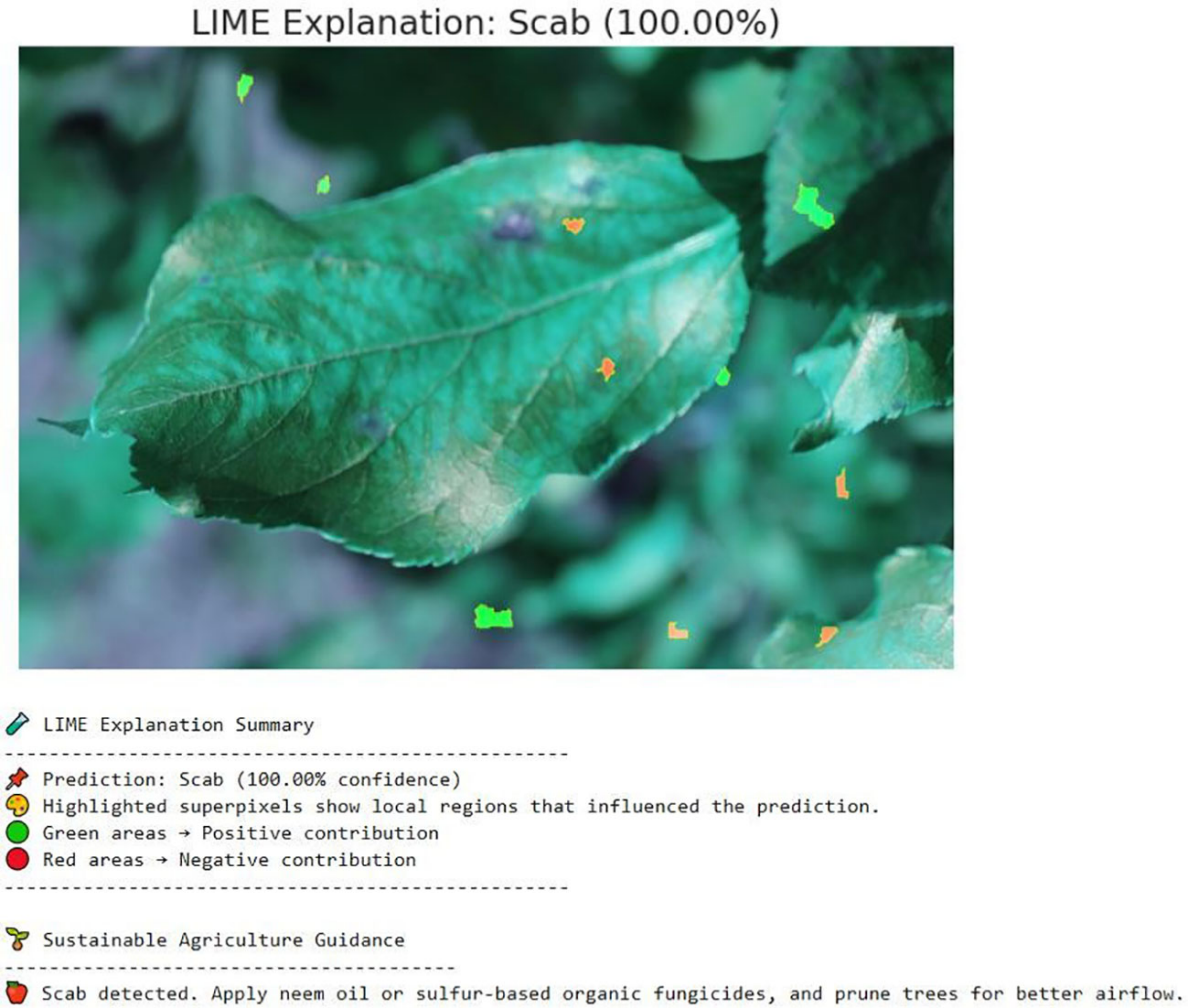
LIME explanation.

The provided study demonstrates the application of LIME (Local Interpretable Model-Agnostic Explanations) to explain the prediction of a deep learning model classifying plant leaf diseases. Specifically, this implementation focuses on interpreting a prediction where the model identifies a leaf as being affected by Scab with 100% confidence, as illustrated in the **Figure 21**. The process begins by selecting and preprocessing an image—resizing it to a standard input shape and normalizing pixel values. This preprocessed image is passed to a LimeImageExplainer, which generates explanations by creating thousands of perturbed versions of the image and analyzing how these changes affect the model’s output. A wrapper function (predict_fn) ensures compatibility by feeding these perturbed samples to the model and returning class probabilities.

The LIME explainer identifies superpixels (contiguous pixel regions) that contribute most to the classification decision. These regions are visualized with green highlighting those that positively influenced the prediction and red indicating negative influence. In the explanation image, multiple green superpixels appear on or around blemishes on the leaf surface—indicating that the model’s decision for Scab is based on visible disease symptoms.

Red areas represent regions that, if altered, might reduce confidence in the Scab diagnosis, suggesting they appear more neutral or healthy.

In addition to interpretability, the code includes a sustainability-oriented module that provides actionable advice based on the predicted disease class. For Scab, the model recommends using neem oil or sulfur-based organic fungicides and pruning trees to improve airflow—highlighting a practical, eco-friendly intervention. Overall, this LIME-based pipeline not only validates that the model’s decisions are grounded in meaningful visual features but also bridges the gap between AI-based diagnostics and real-world sustainable agricultural practices.

## 6 Discussion

The comparative analysis presented in **Table 10** highlights the performance of various state-of-the-art methodologies using accuracy as the evaluation metric. Among the 14 referenced models, the proposed AgriX- SENet achieves the highest accuracy of 0.9747, outperforming all existing methods. The closest competitor, a CNN-based model referenced in (Rahman et al., 2024), attains an accuracy of 0.9665, which is 0.82% lower than AgriX-SENet. Other high-performing models include DLMC-Net ( (Sharma et al., 2023), 0.9656), ShuffleNetV2 ( (Lu L. et al., 2022), 0.9628), and MobileNetV2 ( (Alkanan and Gulzar, 2024), 0.9627). Notably, traditional and hybrid deep learning architectures such as VGG-19-based transfer learning ( (Latif et al., 2022), 0.9608) also fall short, with margins ranging from 1.0% to 1.5% below AgriX-SENet. In the context of high-accuracy tasks, even a 0.5% improvement is considered meaningful, underscoring the significance of AgriX-SENet’s lead.

**Table 10:** Comparative analysis of other state of art methodology.

Ref no	Model	Accuracy
(Rahman et al., 2024)	CNN	.9665
(Alkanan and Gulzar, 2024)	MobileNetV2	.9627
(Ashraf et al., 2023)	CNN	.9300
(Maqsood et al., 2021)	CNN	.8300
(Ramcharan et al., 2017)	CNN Inception V3	.9300
(Zhang et al., 2020)	DCNN	.9330
(Xu et al., 2023)	CNN-RB	.9444
(Lu L. et al., 2022)	ShuffleNetV2	.9628
(Sharma et al., 2023)	DLMC-Net	.9656
(Zhao et al., 2021)	RIC-Net	.9520
(Gonzalez-Huitron et al., 2021)	NasNetMobile	.9200
(Oad et al., 2024)	Ensemble Learning	.9230
(Latif et al., 2022)	VGG-19 based Transfer learning	.9608
Proposed methodology	AgriX-SENet	**.9747**

In the lower accuracy group, models such as CNN-RB ( (Ramcharan et al., 2017), 0.9444), RIC-Net ( (Zhao et al., 2021), 0.9520), and DCNN ( (Zhang et al., 2020), 0.9330) show moderate effectiveness but still lag by 2–4%. More substantial underperformance is observed in models like CNN Inception V3 ( (Ramcharan et al., 2017), 0.9300), NasNetMobile ( (Gonzalez-Huitron et al., 2021), 0.9200), and Ensemble Learning approaches ( (Oad et al., 2024), 0.9230), with differences from AgriX-SENet ranging from 4% to over 5%. The poorest performer in the list is CNN (Maqsood et al., 2021), with an accuracy of just 0.8300, representing a substantial 14.47% drop from the proposed model. When calculating the mean accuracy of all 14 previous models, the average comes out to approximately 0.9382, indicating that AgriX-SENet surpasses the average state-of-the-art performance by a significant margin of 3.65%.

**Figure 22:**
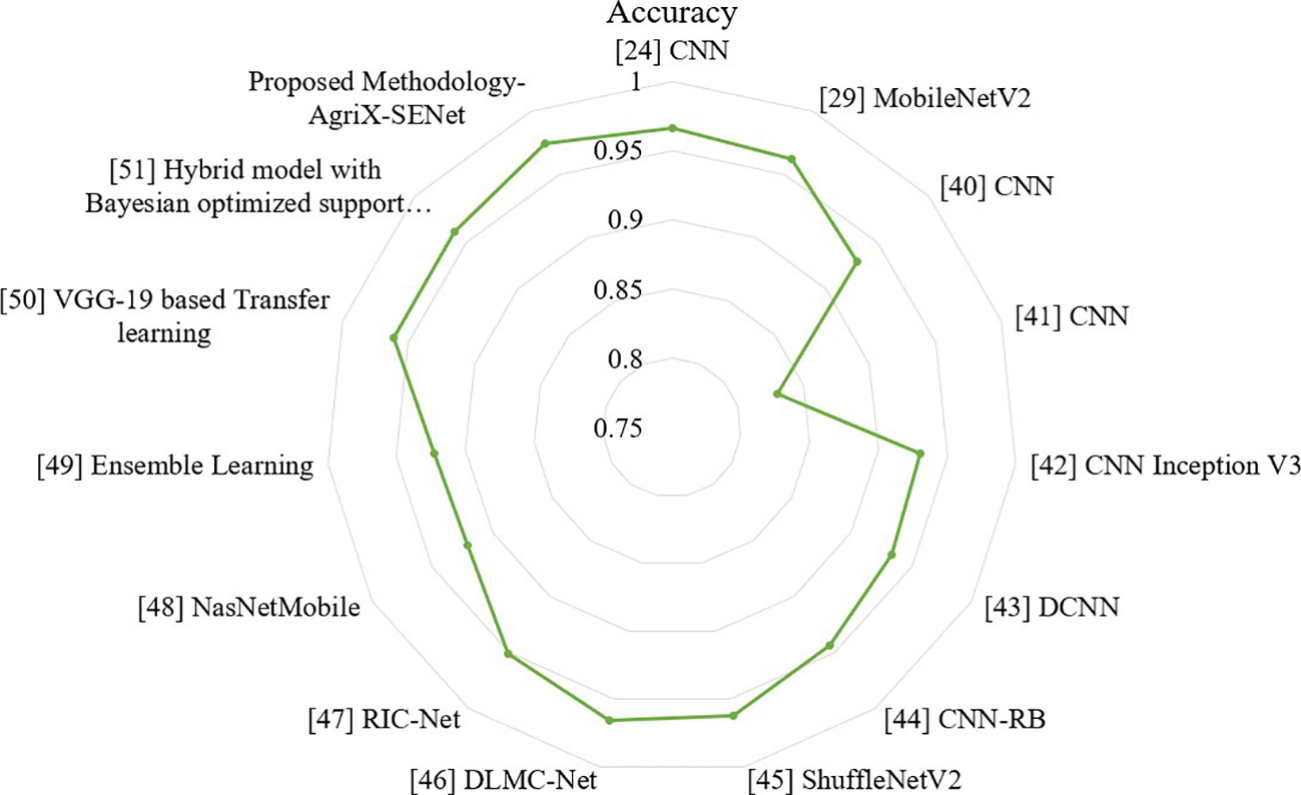
Comparative analysis of other state of art methodology.

This consistent outperformance across a wide spectrum of architectures—from conventional CNNs to mobile- optimized and hybrid models—demonstrates the robustness and efficiency of AgriX-SENet. It suggests that the proposed model likely introduces enhancements in feature extraction, attention mechanisms, or architectural optimization that contribute to its superior accuracy. The analysis strongly supports the conclusion that AgriX- SENet not only leads in raw performance but also sets a new benchmark in the evaluated domain. The comparative analysis is shown in **Figure 22**.

Although AgriX-SENet demonstrates strong performance and achieves superior classification accuracy compared with existing approaches, several limitations should be acknowledged. The framework remains highly dependent on the availability of large, accurately labeled, and diverse datasets. Variations in illumination, viewing angles, background complexity, image quality, and disease severity can influence performance, particularly when such conditions are underrepresented in the training data.

Another challenge lies in the model’s ability to generalize across unseen crop species, rare disease categories, and varying environmental conditions. As a result, additional validation may be required before applying the framework to different agricultural regions or cultivation settings. Furthermore, the incorporation of Squeeze-and-Excitation (SE) modules enhances feature discrimination but introduces additional computational overhead, which may limit deployment on resource-constrained devices without further optimization.

While the proposed framework incorporates explainability through attention mechanisms and complementary XAI techniques, translating these explanations into intuitive insights for non-technical stakeholders remains challenging. The model may also require adaptation or retraining when extended to multi-crop datasets or disease classes with highly overlapping visual symptoms. In addition, the current approach relies solely on static image information and does not exploit complementary contextual factors such as weather conditions, disease progression over time, soil characteristics, or other agronomic variables that could further improve diagnostic accuracy. Finally, the development of high-quality annotated datasets remains labor-intensive and often requires expert knowledge, making large-scale dataset creation both time-consuming and costly.

Despite these limitations, AgriX-SENet offers several important advantages that support its practical value. The framework achieves a classification accuracy of 97.47%, surpassing a range of state-of-the-art methods and demonstrating its effectiveness for plant disease recognition. The integration of SE blocks enables adaptive channel-wise feature refinement, allowing the network to emphasize disease-relevant characteristics while suppressing less informative features. This enhanced representation contributes to improved discrimination of subtle disease symptoms.

The inclusion of explainable AI techniques further strengthens the framework by providing insight into the reasoning behind model predictions, thereby increasing transparency and user confidence. AgriX-SENet also exhibits robust performance across diverse disease manifestations and symptom variations, indicating strong feature-learning capability. Its architecture is compatible with transfer learning strategies, facilitating adaptation to new crops and disease categories with reduced training effort. By enabling accurate and timely disease identification, the framework has the potential to support precision agriculture practices, reduce unnecessary pesticide usage, improve crop productivity, and promote more sustainable farming systems. Moreover, with appropriate optimization techniques such as model compression, pruning, and quantization, the framework can be adapted for deployment on mobile and edge-based agricultural diagnostic platforms.

An important component of the proposed framework is the sustainability-oriented recommendation module, which extends the system beyond disease identification to support practical decision-making. The actionable advice associated with each disease class was curated from established plant disease management practices and widely accepted agricultural recommendations reported in the literature. For example, when Scab is detected, the framework recommends environmentally friendly interventions such as neem oil applications, sulfur-based organic fungicides, and pruning practices that improve airflow and reduce disease spread. These recommendations are intended to provide preliminary guidance rather than replace professional agronomic expertise. By linking disease diagnosis with sustainable intervention strategies, the framework helps bridge the gap between AI-based disease recognition and practical field-level disease management, thereby enhancing its potential utility in sustainable agriculture.

## 7 Conclusion

This study presents AgriX-SENet, a Squeeze-and-Excitation-based deep learning framework for explainable plant disease detection aligned with the goals of sustainable agriculture. The model significantly outperforms existing state-of-the-art methods, achieving a training accuracy of.9747 and a validation accuracy of.9507, highlighting its high precision and generalization capability. Through the integration of attention mechanisms and interpretability tools like Grad-CAM, SHAP, and LIME, the model offers transparency in decision-making, addressing the critical need for explainable AI in agricultural diagnostics. The framework’s ability to emphasize disease-relevant features and its compatibility with edge devices make it suitable for deployment in real-world, resource-constrained agricultural environments. Moreover, the model supports sustainable farming by enabling early detection, reducing chemical usage, and facilitating precision interventions. While the approach demonstrates substantial advantages, it also has limitations such as high data dependency and limited generalizability across unseen diseases or crop types. These areas present opportunities for future research, including expanding the model’s adaptability and incorporating multimodal or temporal data to improve robustness. In the future, we aim to develop a hardware for implementing the proposed architecture physical in an Internet of Things-based scenario.

## Data Availability

The original contributions presented in the study are included in the article/supplementary material. Further inquiries can be directed to the corresponding authors.
